# Supplementary material for: Combinatorial Smad2/3 Activities Downstream of Nodal Signaling Maintain Embryonic/Extra-Embryonic Cell Identities during Lineage Priming
Source: Cell Rep. 2018 Aug 24;24(8):1977–1985.e7. doi: 10.1016/j.celrep.2018.07.077 (PMC6113931; doi:10.1016/j.celrep.2018.07.077)
Supplement: Document S1. Figures S1–S3 and Table S4 [file mmc1.pdf]

Cell Reports, Volume *24*

Supplemental Information

Combinatorial Smad2/3 Activities Downstream of Nodal Signaling Maintain Embryonic/Extra-Embryonic Cell Identities during Lineage Priming

Anna D. Senft, Ita Costello, Hamish W. King, Arne W. Mould, Elizabeth K. Bikoff, and Elizabeth J. Robertson

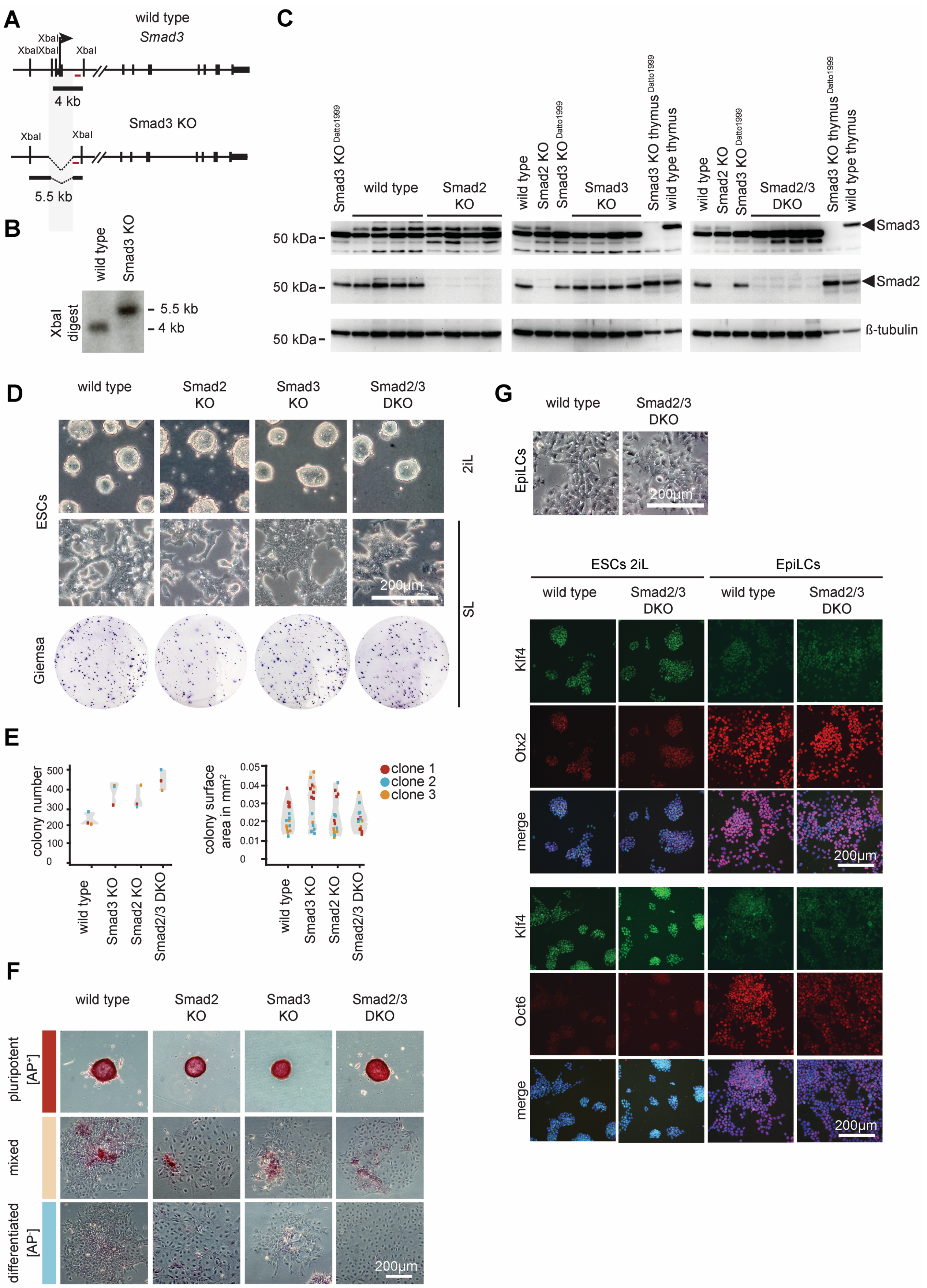

Figure S1
related to Figure 1

Generation and characterization of Smad2/3 DKO, Smad3 KO and Smad2 KO ESCs and Smad2/3 DKO EpiLCs

A. Smad2/3 DKO and Smad3 KO ESC lines were obtained by targeting Smad2 KO and WT ESC lines using the *Smad3* deletion strategy shown. The shaded area indicates the genomic region of mouse *Smad3* around exon 1 removed by CRISPR/Cas9 nickase technology. Red bar and thin black lines depict the 3' Southern probe and XbaI restriction sites used for genotyping the deletion in (B). See Key Resources Table and Table S4 for sequences for sgRNAs and Southern probe.
B. Southern blot to screen for $Smad3^{\Delta exon1/\Delta exon1}$ and Smad2 KO;$Smad3^{\Delta ex1/\Delta ex1}$ (hereafter referred to as Smad3 KO and Smad2/3 DKO) ESC lines with the 3' probe depicted in (A) on XbaI-digested genomic DNA. Homozygous targeted clones contain a single band at 5.5 kb compared to 4 kb in WT *Smad3*.
C. Western blot of Smad2/3 DKO, Smad3 KO as well as parental Smad2 KO and WT ESC lines used in study with antibodies specific to Smad3, Smad2 and β-tubulin. The recognition site of the Smad3 antibody lies in the region most divergent between the highly homologous Smad2 and Smad3 proteins and is - on the genomic level - unaffected by the deletion strategy used. Conventionally derived $Smad3^{tm1Xfw/tm1Xfw}$ ESCs as well as thymus isolated from $Smad3^{tm1Xfw/tm1Xfw}$ male mice (Datto et al. 1999) were used as controls. Note that prominent bands in Smad3 blots represent unspecific background that is present in all ESC samples but absent in thymus lysates.
D. Morphology of WT, Smad2 KO, Smad3 KO and Smad2/3 DKO ESCs grown under 2iL and SL conditions. Giemsa staining of ESC colonies grown for 7 days following plating of 675 ESCs (SL).
E. Violin plots depicting colony number from cultures of three independent ESC lines per genotype (left) and colony surface area of 5 colonies for each independent ESC line (right).
F. Representative images of pluripotent (red), mixed (beige) and differentiated (blue) colonies after alkaline phosphatase (AP) staining on WT, Smad2 KO, Smad3 KO and Smad2/3 DKO ESC colonies (SL) grown for five days in the presence or absence of LIF. Related to Figure 1C.
G. Brightfield images and immunofluorescence staining of WT and Smad2/3 DKO ESCs (2i) and EpiLCs using Klf4 together with Otx2 or Oct6-specific antibodies and counterstained with DAPI.

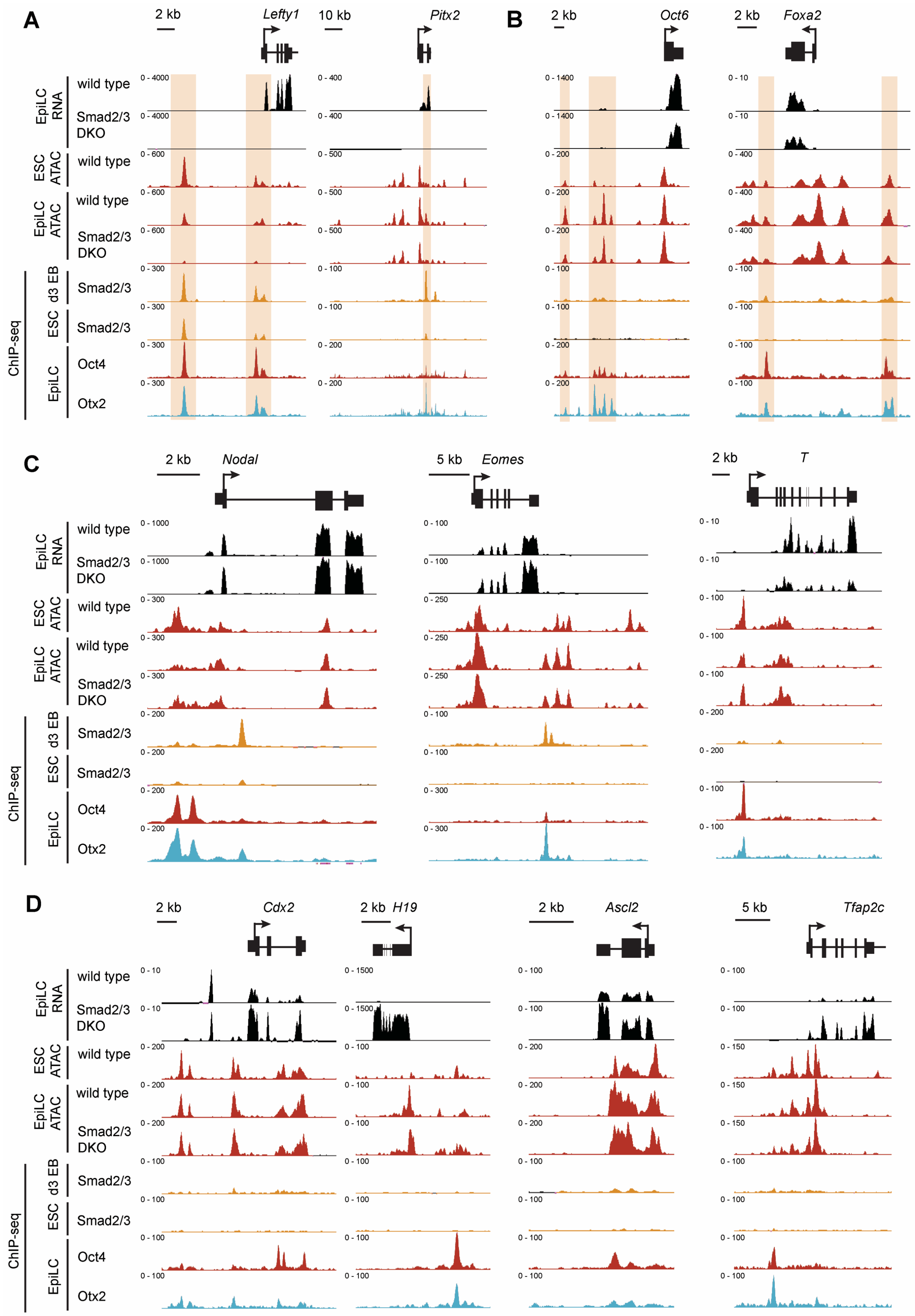

Figure S2
related to Figure 2

Smad2/3 alters chromatin accessibility at Nodal-dependent and priming marker genes but not at embryonic and extra-embryonic differentiation genes in EpiLCs

A-D. Genome browser screenshots of RNA-seq and ATAC-seq tracks in Smad2/3 DKO and WT EpiLCs at loci of Nodal target genes (A), primed pluripotency genes (B), early differentiation markers (C) and extra-embryonic marker genes (D). ChIP-seq tracks of Smad2/3 occupancy in ESCs and d3 EBs (Wang et al. 2017) and Oct4 and Otx2 occupancy in EpiLCs (Buecker et al., 2014) and ATAC-seq signal in ESCs (Simon et al., 2017) (D) are also shown.

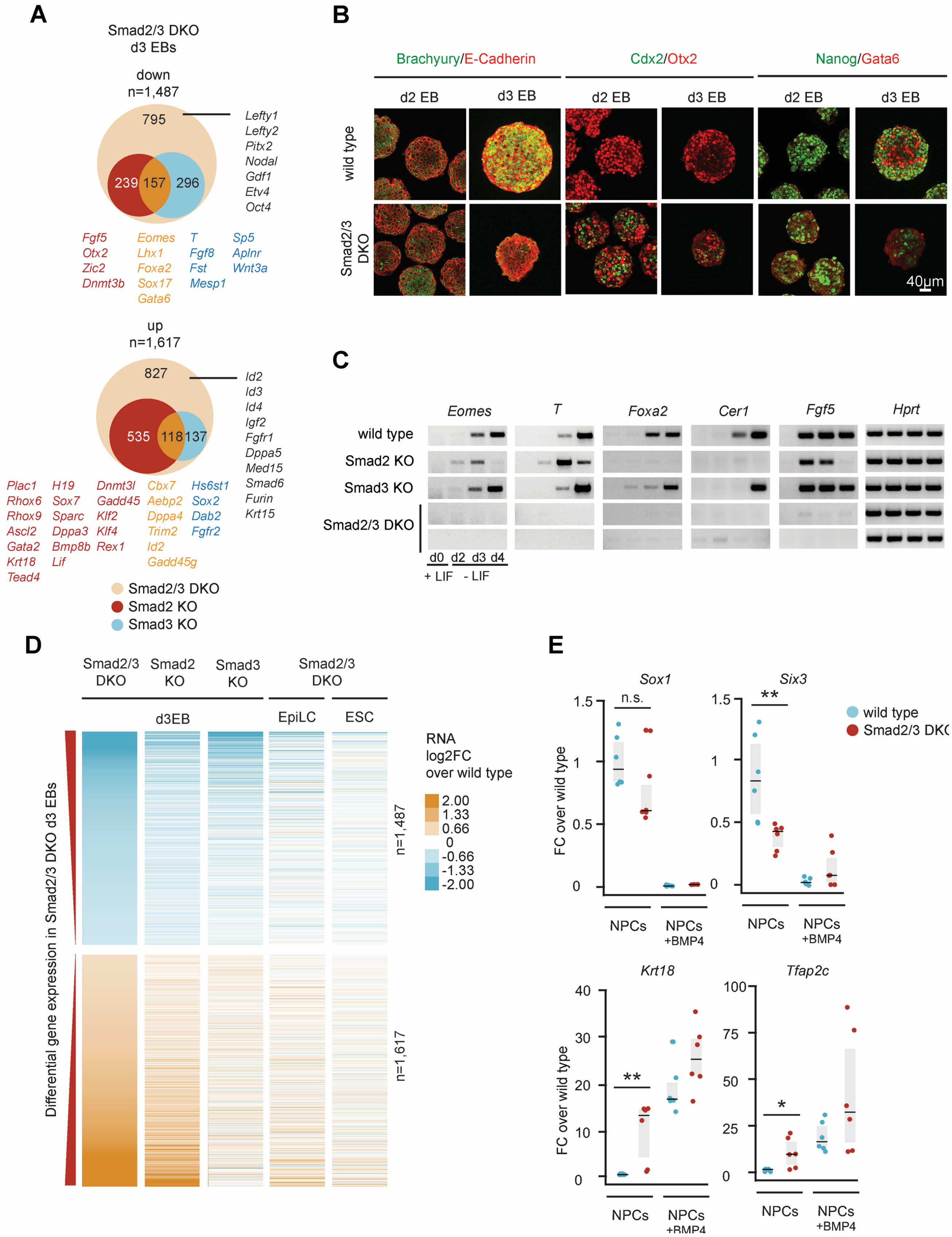

Figure S3
related to Figure 3

Combinatorial Smad2 and Smad3 activities mediate embryonic cell fate allocation and repression of extra-embryonic gene expression

A. Venn diagrams showing the overlap between significant changes in gene expression in Smad2 KO, Smad3 KO and Smad2/3 DKO d3 EBs relative to WT d3 EBs as determined by microarray profiling (n=4). Genes unique to Smad2 KO or Smad3 KO EBs were omitted. A list of all deregulated genes is shown in Table S1.
B. Immunofluorescence staining of WT and Smad2/3 DKO d2 and d3 EBs with antibodies specific to Brachyury and E-Cadherin (left), Otx2 and Cdx2 (centre) and Nanog and Gata6 (right).
C. Validation of reduced gene expression of selected down-regulated genes during EB differentiation of WT, Smad2 KO, Smad3 KO and Smad2/3 DKO ESCs by OneStep RT-PCR. *Hprt* expression is used as a control.
D. Heatmap showing the log2FC in expression of differentially expressed genes in Smad2/3 DKO d3 EBs as compared to WT d3 EBs and their expression in Smad2 KO and Smad3 KO d3 EBs, Smad2/3 DKO EpiLCs and ESCs relative to their respective WT controls.
E. qRT-PCR measurement of gene expression changes in Smad2/3 DKO d5 NPCs (n=3) grown in the absence or presence of BMP4 (5ng/ml) using primers specific to *Sox1*, *Six3*, *Krt18* and *Tfap2c* (encoding AP2γ) normalized to *Gapdh* expression. *$p<0.05$, **$p<0.01$.

Table S4 related to Figures S1 and S3

List of primers used in this study including PCR and Southern probe genotyping primers, OneStep RT-PCR and qRT-PCR primers.

Category / Gene	forward	reverse
Genotyping		
Smad3 tm1Xfw	CTCCAGAGTTAAAAGCGAAGTTCG	rev1: AAAATGCTGCACGGAAGCCAGGTC<br>rev2: ATTTGTCACGTCCTGCACGACG
Smad2 Robm1	TATTGCTGAAGAGCTTGGCG	rev1: TGGCCTTAGAAAAGTTAATTGCCC<br>rev2: GCAAACCACAGCACACAACA
Southern blot probe		
Smad3 3' exon 1	CAGGCGATGGTTCTGTCCTT	GCCAGGGCAGGATTCAAGAT
OneStep RT-PCR		
Eomes	TGTTTTCGTGGAAGTGGTTCTGGC	AGGTCTGAGTCTTGGAAGGTTCATTC
T	AACTTTCCTCCATGTGCTGAGAC	TGACTTCCCAACACAAAAAGCT
Foxa2	TGGCTGCAGACACTTCCTACT	CAACATCAGTACAACCCTCTGGT
Cer1	GGAGGAAGCCAAGAGGTTCT	GTCTTCATGGGCAATGGTCT
Fgf5	TTGCGACCCAGGAGCTTAAT	CTACGCCTCTTTATTGCAGC
Hprt	GCTGGTGAAAAGGACCTCT	CACAGGACTAGAACACCTGC
qRT-PCR		
Sox1	GTGACATCTGCCCCCATC	GAGGCCAGTCTGGTGTCAG
Six3	CCGGAAGAGTTGTCCATGTTC	CGACTCGTGTTTGTTGATGGC
Krt18	CAGCCAGCGTCTATGCAGG	CTTTCTCGGTCTGGATTCCAC
Tfap2c	GCCGGACGCCATGTTGTGGA	ACCCCGGTGTGCGAGAGAGG
Gapdh	TGCACCACCAACTGCTTAGC	GGCATGGACTGTGGTCATGAG
