# Supplementary material for: Combinatorial Smad2/3 Activities Downstream of Nodal Signaling Maintain Embryonic/Extra-Embryonic Cell Identities during Lineage Priming
Source: Cell Rep. 2018 Aug 24;24(8):1977–1985.e7. doi: 10.1016/j.celrep.2018.07.077 (PMC6113931; doi:10.1016/j.celrep.2018.07.077)
Supplement: Document S2. Article plus Supplemental Information [file mmc5.pdf]

Cell Reports

Report

Combinatorial Smad2/3 Activities Downstream of Nodal Signaling Maintain Embryonic/Extra-Embryonic Cell Identities during Lineage Priming

Graphical Abstract

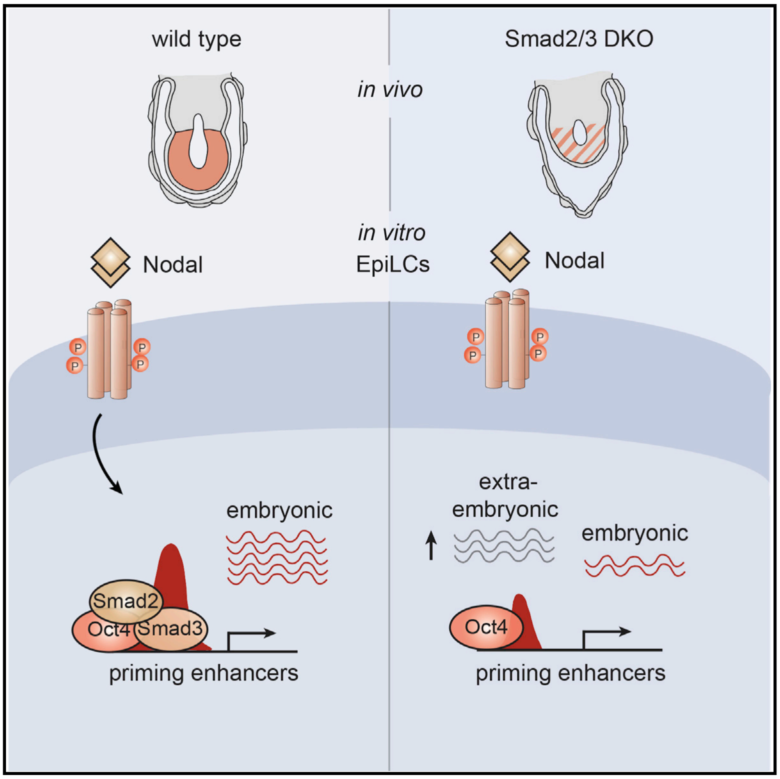

Highlights

- Smad2/3 alters the transcriptome and activity of distal regulatory elements in EpiLCs
- Smad2 prevents expression of extra-embryonic genes during priming and differentiation
- Smad2/3 is essential for mesoderm and definitive endoderm cell fate allocation
- Smad2/3 signaling balances Bmp signaling during neural precursor differentiation

Authors

Anna D. Senft, Ita Costello, Hamish W. King, Arne W. Mould, Elizabeth K. Bikoff, Elizabeth J. Robertson

Correspondence

elizabeth.robertson@path.ox.ac.uk

In Brief

Using genetic ablation of Smad2/3 effectors downstream of Nodal signaling in mouse embryonic stem cells, Senft et al. show a requirement for Smad2/3 signaling to maintain embryonic/extra-embryonic cell identities during lineage priming before mesoderm and endoderm cell fate allocation and to balance Nodal/Bmp signaling during ectoderm differentiation.

Senft et al., 2018, Cell Reports *24*, 1977–1985
August 21, 2018 © 2018 The Author(s).
https://doi.org/10.1016/j.celrep.2018.07.077

CellPress

OPEN ACCESS
CellPress

Combinatorial Smad2/3 Activities Downstream of Nodal Signaling Maintain Embryonic/Extra-Embryonic Cell Identities during Lineage Priming

Anna D. Senft,[1] Ita Costello,[1] Hamish W. King,[2] Arne W. Mould,[1] Elizabeth K. Bikoff,[1] and Elizabeth J. Robertson[1,3,*]
[1]Sir William Dunn School of Pathology, University of Oxford, Oxford OX1 3RE, UK
[2]Department of Biochemistry, University of Oxford, Oxford OX1 3QU, UK
[3]Lead Contact
*Correspondence: elizabeth.robertson@path.ox.ac.uk
https://doi.org/10.1016/j.celrep.2018.07.077

SUMMARY

Epiblast cells in the early post-implantation stage mammalian embryo undergo a transition described as lineage priming before cell fate allocation, but signaling pathways acting upstream remain ill defined. Genetic studies demonstrate that Smad2/3 double-mutant mouse embryos die shortly after implantation. To learn more about the molecular disturbances underlying this abrupt failure, here we characterized Smad2/3-deficient embryonic stem cells (ESCs). We found that Smad2/3 double-knockout ESCs induced to form epiblast-like cells (EpiLCs) display changes in naive and primed pluripotency marker gene expression, associated with the disruption of Oct4-bound distal regulatory elements. In the absence of Smad2/3, we observed enhanced Bmp target gene expression and de-repression of extra-embryonic gene expression. Cell fate allocation into all three embryonic germ layers is disrupted. Collectively, these experiments demonstrate that combinatorial Smad2/3 functional activities are required to maintain distinct embryonic and/or extra-embryonic cell identity during lineage priming in the epiblast before gastrulation.

INTRODUCTION

The strict segregation of embryonic and extra-embryonic tissues constitutes the earliest cell fate decision in the pre-implantation mammalian embryo. Later, during gastrulation through coordinated patterning by Nodal and Bmp signaling, pluripotent epiblast cells are induced to form the three primary germ layers: mesoderm, definitive endoderm (DE), and ectoderm (Robertson, 2014). However, studies demonstrate that epiblast cells acquire competence to differentiate in response to inductive signaling cues at earlier stages (Smith, 2017). This cellular transition, designated as lineage priming or epiblast maturation and characterized in cultured epiblast-like cells (EpiLCs) (Buecker et al., 2014; Hayashi et al., 2011), is associated with genome-wide reorganization of active enhancers, resulting in decreased expression of naive pluripotency genes, activation of primed and early differentiation genes, and importantly, stable repression of extra-embryonic gene expression (Morgani et al., 2017; Murakami et al., 2016). However, relatively little is known about the underlying molecular mechanisms driving these cellular events.

Our early work showed that Nodal, a member of the transforming growth factor β (TGF-β) superfamily of secreted growth factors, is required for axis patterning in the early post-implantation stage embryo (Brennan et al., 2001). Loss-of-function embryos arrest before gastrulation, fail to form mesoderm, prematurely lose expression of pluripotency markers, and precociously activate neuroectoderm markers (Brennan et al., 2001; Camus et al., 2006; Mesnard et al., 2006). Nodal receptors activate the closely related downstream intracellular effectors Smad2 and Smad3 (Smad2/3) that translocate into the nucleus to regulate target gene expression (Massagué, 2012). Smad2/3 share >90% amino acid identity and display partially overlapping expression patterns in the early embryo (Dunn et al., 2004; Waldrip et al., 1998). However, loss-of-function mutants display strikingly different phenotypes. Animals lacking Smad3 are adult viable (Datto et al., 1999). In contrast, *Smad2* mutant embryos fail to acquire anterior-posterior patterning and arrest shortly after implantation (Waldrip et al., 1998). Instead, because of loss of Smad2 in the extra-embryonic primitive endoderm (where Smad3 is not expressed), the epiblast defaults exclusively to an extra-embryonic mesodermal fate (Dunn et al., 2004; Waldrip et al., 1998). *Smad3* expression from the *Smad2* locus can rescue the lethal phenotype (Dunn et al., 2005). Moreover, Smad2/3 clearly function in a dose-dependent manner (Vincent et al., 2003). Thus, double-mutant embryos lacking both Smad2/3 abruptly arrest shortly after implantation and are severely disorganized (Dunn et al., 2004).

Previous efforts aimed at dissecting partially overlapping Smad2/3 functional contributions have been hampered by this early lethality. Here we exploited embryonic stem cells (ESCs) lacking both Smad2/3, in combination with directed *in vitro* differentiation protocols, to gain insight into the underlying defects. We observe that Smad2/3 double-mutant ESCs fail to undergo lineage priming or correct cell fate allocation and ectopically activate extra-embryonic genes. This priming defect was associated with inappropriate

This is an open access article under the CC BY license (http://creativecommons.org/licenses/by/4.0/).

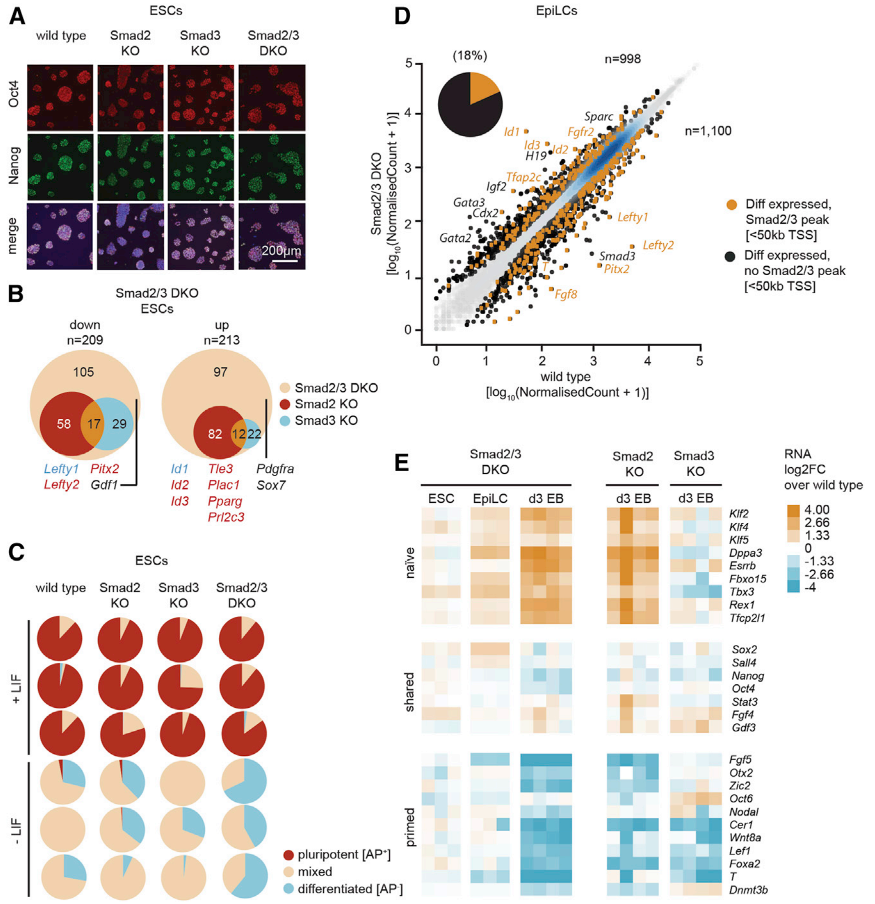

Figure 1. Smad2/3 Repress Expression of Extra-Embryonic and Naive Pluripotency Genes during Lineage Priming

(A) WT, Smad2 KO, Smad3 KO, or Smad2/3 DKO ESCs (2iL) were stained for Oct4 and Nanog and counterstained with DAPI.

(B) Venn diagrams showing significant changes in gene expression shared by Smad2 KO, Smad3 KO, and Smad2/3 DKO ESCs, relative to WT ESCs, as determined by microarray profiling (n = 3 or 4). Genes uniquely differentially expressed by Smad2 KO or Smad3 KO ESCs were excluded from this analysis. A summary of deregulated genes is presented in Table S1.

(C) Pie charts of alkaline phosphatase (AP)-stained WT, Smad2 KO, Smad3 KO, or Smad2/3 DKO ESCs cultured for 5 days in the presence or absence of LIF (n = 3), corresponding to pluripotent, differentiated, or mixed colonies. See also Figure S1F.

(D) Scatterplot showing significantly ($p < 0.05$, Benjamini-Hochberg adjusted) differentially expressed genes in Smad2/3 DKO EpiLCs compared to WT by RNA-seq (n = 3). The cutoff was set to >1.5-fold change. Differentially expressed genes near Smad2/3 ChIP-seq peaks in day 3 EBs ($\leq$50 kb of its transcriptional start site [TSS]) are indicated in yellow. The pie chart indicates the proportion of differentially expressed genes also showing Smad2/3 ChIP-seq peaks ($\leq$50 kb of TSS).

(E) Heatmap showing relative expression levels of pluripotency marker genes in Smad2/3 DKO ESCs, EpiLCs, and day 3 EBs compared to WT controls (n = 3 or 4). Profiles of Smad2 KO and Smad3 KO day 3 EBs are shown on the right.

activation of Oct4-bound distal regulatory sites and enhanced Bmp target gene expression. Collectively, the present results demonstrate that combinatorial Smad2/3 activities are required to maintain embryonic identity in the early epiblast during lineage priming.

RESULTS

Smad2/3 Inactivation in ESCs Fails to Disrupt Self-Renewal and Expression of Pluripotency Marker Genes

To investigate functional contributions made by the closely related Smad2/3 transcriptional regulators, we targeted the *Smad3* promoter in $Smad2^{-/-}$ (Smad2 knockout [KO]) (Tremblay et al., 2000) or wild-type (WT) ESCs to generate $Smad2^{-/-};Smad3^{-/-}$ ESCs (Smad2/3 double knockout [DKO]) and $Smad3^{-/-}$ ESCs (Smad3 KO) (Figure S1A). Correctly targeted clones were identified by Southern blot analysis, and loss of Smad3 protein expression was confirmed by western blotting (Figures S1B and S1C). WT, Smad2 KO, Smad3 KO, and Smad2/3 DKO ESCs cultured under 2i + LIF (2iL) or serum + LIF (SL) conditions were morphologically indistinguishable (Figure S1D). Smad2/3 DKO ESCs efficiently formed colonies and displayed robust Oct4 and Nanog expression levels (Figures 1A, S1D, and S1E).

To examine gene expression changes, we carried out transcriptional profiling experiments using microarrays. We identified 422 genes with significantly changed expression levels (209 down and 213 up) in Smad2/3 DKO ESCs cultured under SL conditions compared to WT ESCs (Figure 1B; Table S1). Several Nodal targets (*Lefty1/2* and *Pitx2*) were downregulated, while Bmp target genes (*Id1/2/3)* were upregulated. Genes normally confined to trophectoderm derivatives (*Tle3*, *Plac1*, *Pparg*, and *Prl2c3*) and extra-embryonic primitive endoderm (*Pdgfra* and *Sox7*) were upregulated in Smad2/3 DKO ESCs. A subset of differentially expressed genes was also significantly altered in either Smad2 KO or Smad3 KO ESCs (33% and 12%, respectively) (Figure 1B). Smad2, but not Smad3, KO ESCs display upregulated extra-embryonic gene expression. There was no evidence for altered expression of pluripotency markers (Table S1).

Loss of Smad2/3 Results in Activation of Extra-Embryonic and Bmp Target Gene Expression upon Exit from the Undifferentiated State

To test whether these transcriptional changes potentially influence exit from the naive state, we induced differentiation by plating ESCs (SL) at low density in the absence of LIF and performed alkaline phosphatase (AP) staining to identify naive ESCs. Similar to WT or single-KO ESCs, Smad2/3 DKO ESCs gave rise to AP-negative colonies (Figure 1C). As for

BMP4-treated WT ESCs that similarly exhibit a bias toward extra-embryonic gene expression, Smad2/3 DKO and to a lesser extent Smad2 KO ESC colonies displayed a distinctive, more flattened, epithelial morphology (Hayashi et al., 2010) (Figure S1F).

To further explore exit from the naive state, we compared gene expression profiles of WT and Smad2/3 DKO EpiLCs using RNA sequencing (RNA-seq). As expected, WT ESCs (2iL) induced to form EpiLCs by activin A and Fgf2 treatment adopted a flattened cell morphology. The appearance of Smad2/3 DKO EpiLCs closely resembled WT (Figure S1G). RNA-seq analysis identified 2,098 genes with significantly changed expression in Smad2/3 DKO compared to WT EpiLCs (1,100 down and 998 up) (Figure 1D; Table S2). We found that expression levels of Nodal-dependent genes (e.g., *Lefty1/2* and *Pitx2*) and early mesoderm markers (e.g., *T* and *Fgf8*) were downregulated in Smad2/3 DKO EpiLCs. However, Bmp targets (e.g., *Id1*/2/3/4) and extra-embryonic ectoderm (e.g., *Fgfr2* and *Tfap2c*), trophectoderm (e.g., *Gata2* and *Gata3*), and visceral endoderm (VE) (e.g., *H19* and *Sparc*) marker genes were activated.

Next, we compared the list of differentially expressed genes with published Smad2/3 chromatin immunoprecipitation sequencing (ChIP-seq) datasets (Wang et al., 2017). We found that 18% of differentially expressed genes in Smad2/3 DKO EpiLCs were occupied by Smad2/3 in differentiated embryoid bodies (EBs), in contrast to only 3% in ESCs (Table S2). Smad2/3-occupied differentially expressed genes included both Nodal and Bmp targets. Except for *Fgfr2* and *Tfap2c* (encoding Ap2γ) none of the ectopically activated extra-embryonic genes were found to be Smad2/3 occupied, which implicates an indirect regulatory mechanism, assuming Smad2/3 occupancy is similar between EpiLCs and EBs.

Combinatorial Smad2/3 Activities Control Pluripotency-Associated Gene Expression during Lineage Priming

Smad2/3 DKO EpiLCs display enhanced expression of naive pluripotency markers (e.g., *Klf2* and *Rex1*) characteristic of the blastocyst inner cell mass, together with decreased expression of primed pluripotency markers (e.g., *Fgf5* and *Oct6*) (Figure 1E). Reduced Oct6 expression was confirmed by immunofluorescence staining experiments (Figure S1G). In contrast, expression of the epiblast marker *Otx2* was unchanged (Figures 1E and S1G). To further examine gene expression changes during differentiation, Smad2/3 DKO ESCs were induced to form EBs and transcriptional profiles were analyzed using microarrays. As shown in Figure 1E, Smad2/3 DKO EBs display striking downregulation of primed pluripotency markers and upregulated expression of naive pluripotency genes. In addition, when we analyzed single-KO EBs, we found that Smad2 KO EBs, but not Smad3 KO EBs, resembled Smad2/3 DKO EBs. Consistent with this, EpiLCs express *Smad2* at roughly 5-fold higher levels in comparison with *Smad3*. Thus, Smad2 activity is predominantly responsible for governing the gain of primed and/or differentiated identity during lineage priming.

Smad2/3 Influence Chromatin Accessibility during the ESC-to-EpiLC Transition

The preceding results demonstrate that Smad2/3 activities regulate pluripotency-associated gene expression. However, we detected a relatively low degree of overlap between Smad2/3-dependent transcripts and nearby sites of Smad2/3 binding using published Smad2/3 ChIP-seq datasets from day 3 EBs (Wang et al., 2017). These experimental approaches provided only a limited view of the Smad2/3 regulatory network. Global changes in the activities of distal regulatory elements, such as enhancers, during acquisition of the primed state have been previously documented (Buecker et al., 2014; Factor et al., 2014). Therefore, we decided to evaluate possible changes affecting the distal regulatory element landscape in Smad2/3 DKO EpiLCs using the assay for transposase accessible chromatin (ATAC)-seq.

We identified 4,274 regulatory elements that were differentially accessible in Smad2/3 DKO compared to WT EpiLCs (3,234 decreased and 1,040 increased) (Figure 2A; Table S3). To explore the possibility that Smad2/3-dependent regulatory elements potentially influence the transition to the primed state, we identified sites where chromatin accessibility is changed during the ESC-to-EpiLC transition by comparing EpiLC ATAC-seq data with our published WT ESC ATAC-seq dataset (Simon et al., 2017). Most (90%) Smad2/3-dependent sites undergo changes in chromatin accessibility during the ESC-to-EpiLC transition (Figure 2B). Furthermore, many of these were located more than 5 kb from transcriptional start sites (Figure 2C) and displayed an enrichment for markers of distal regulatory elements, including p300, H3K27ac, and H3K4me1 (Figure 2D). These observations suggest that combinatorial Smad2/3 activities influence the global reconfiguration of the chromatin landscape during lineage priming.

Changes in chromatin accessibility at distal regulatory elements in Smad2/3 DKO EpiLCs potentially influence expression of nearby genes. To test this possibility, we compared ATAC-seq and transcriptional profiles. We found that sites with decreased accessibility were associated with reduced expression of nearby genes (Figure 2E). For example, *Lefty1/2* and *Pitx2* display decreased chromatin accessibility at nearby enhancer regions normally occupied by Smad2/3 during differentiation (Figures 2F, 2G, and S2A). In contrast, sites that displayed increased chromatin accessibility were associated with increased expression levels (Figure 2E); however, unlike sites showing decreased accessibility, these loci tended to have lower levels of Smad2/3 binding (Figure 2G). Sites of increased accessibility are associated with increased Smad1 occupancy in BMP4-treated ESCs (Morikawa et al., 2016) (Figure 2G). Collectively, these results demonstrate that Smad2/3 act to promote full access to distal regulatory elements governing gene expression changes necessary for cells to transition to the primed state.

Oct4 and Otx2 Occupy Smad2/3-Dependent Distal Regulatory Elements

Studies demonstrate that Oct4 binding switches from naive enhancers to primed enhancers at pluripotency genes coincident with occupancy by so-called mediators of the primed state, such as Otx2, during the ESC-to-EpiLC transition (Buecker et al., 2014). Here we observe in EpiLCs an enrichment of Oct4 and Otx2 ChIP-seq signals at Smad2/3-dependent sites that display decreased chromatin accessibility (Figures 2F and 2G). Motif enrichment analysis confirmed these sites are

OPEN
ACCESS
CellPress

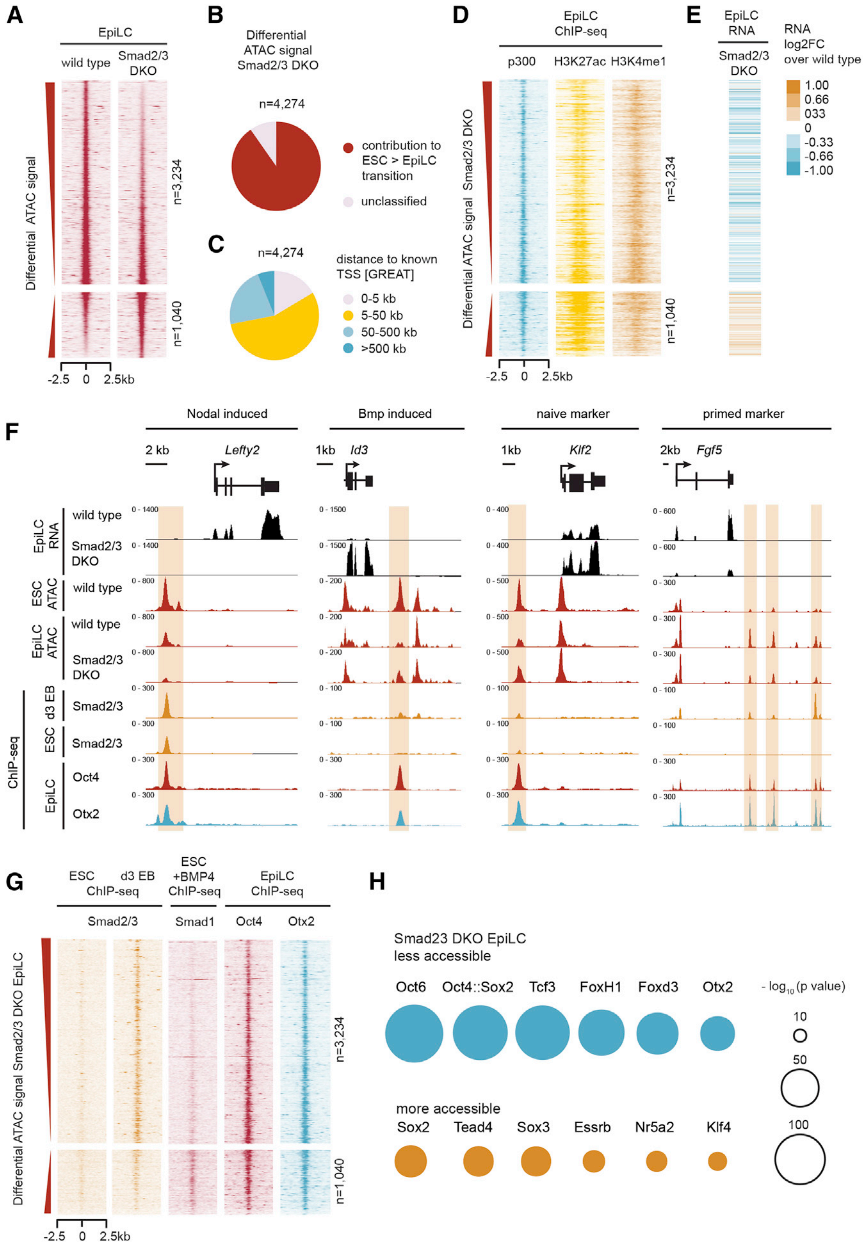

Figure 2. Smad2/3 Influences the Activity of Oct4-Occupied Distal Regulatory Enhancers during Priming

(A) Heatmap of regulatory elements with differential chromatin accessibility in Smad2/3 DKO EpiLCs compared to WT EpiLCs, as measured by ATAC-seq (false discovery rate [FDR] < 0.05, fold change > 1.5). Sites with decreased chromatin accessibility (top) and sites with increased chromatin accessibility (bottom) are ranked by decreasing and increasing ATAC signal change. See also Table S3.

(B and C) Pie charts indicating (B) distributions of differential accessible sites in Smad2/3 DKO EpiLCs compared to WT overlapping with regulatory elements gained or lost during the ESC-to-EpiLC transition or (C) the distance to known TSS as defined by Genomic Regions Enrichment of Annotations Tool (GREAT).

(D) Heatmap read density plots of p300, H3K27ac, and H3K4me1 ChIP-seq signal at regulatory elements with differential accessibility in Smad2/3 DKO EpiLCs (ranked as in A).

(E) Heatmap depicting the log2 fold change (log2FC) in gene expression in Smad2/3 DKO EpiLCs relative to WT EpiLCs as determined by RNA-seq. Genes nearest regulatory elements with differential accessibility in Smad2/3 DKO EpiLCs are shown.

(F) Genome browser snapshots of RNA-seq and ATAC-seq tracks in Smad2/3 DKO and WT EpiLCs at selected genomic loci. ATAC-seq of WT ESCs, ChIP-seq tracks of Smad2/3 occupancy in ESCs and day 3 EBs, and Oct4 and Otx2 occupancy in EpiLCs are also shown.

(G) Heatmap read density plots of WT Smad2/3, Smad1, Oct4, and Otx2 ChIP-seq signal in the indicated cell types at regulatory elements with differential accessibility in Smad2/3 DKO EpiLCs (ranked as in A). ESCs were treated with 10 ng/mL BMP4 for Smad1 ChIP-seq.

(H) Motif enrichment analysis of regulatory elements with differential chromatin accessibility in Smad2/3 DKO EpiLCs. Motifs for transcription factors associated with primed or naive, extra-embryonic, and neural cell states were significantly enriched.

highly enriched for Oct4 and Otx2 binding motifs and those recognized by other priming factors, e.g., Oct6 and Foxd3 (Figure 2H). *Fgf5*, *Oct6*, and *Foxa2*, genes characteristic of the primed state, display decreased accessibility (Figures 2F and S2B). In contrast, in the absence of Smad2/3, *Klf2*, an Oct4/Otx2-occupied gene that characterizes the naive state, shows increased accessibility at a neighboring distal regulatory element and increased expression levels (Figure 2F). These genomic regions were also enriched for naive, extra-embryonic, and neural transcription factor binding motifs (Figure 2H). However, accessibility near early differentiation and extra-embryonic marker genes appeared to be largely unaffected (Figures S2C and S2D). These observations strengthen the argument that cooperative binding by different transcription factors is required during lineage priming and demonstrate that Smad2/3, together with the pioneer factor Oct4 (Mullen et al., 2011; Ruetz et al., 2017), targets distal regulatory elements controlling the transition from the naive to the primed state.

Loss of Smad2/3 Disrupts Cell Fate Allocation during ESC Differentiation

To investigate downstream consequences resulting from this priming defect, we re-examined the Smad2/3 DKO EB microarray profiles. We identified 3,104 genes showing significantly changed expression levels in Smad2/3 DKO compared to WT

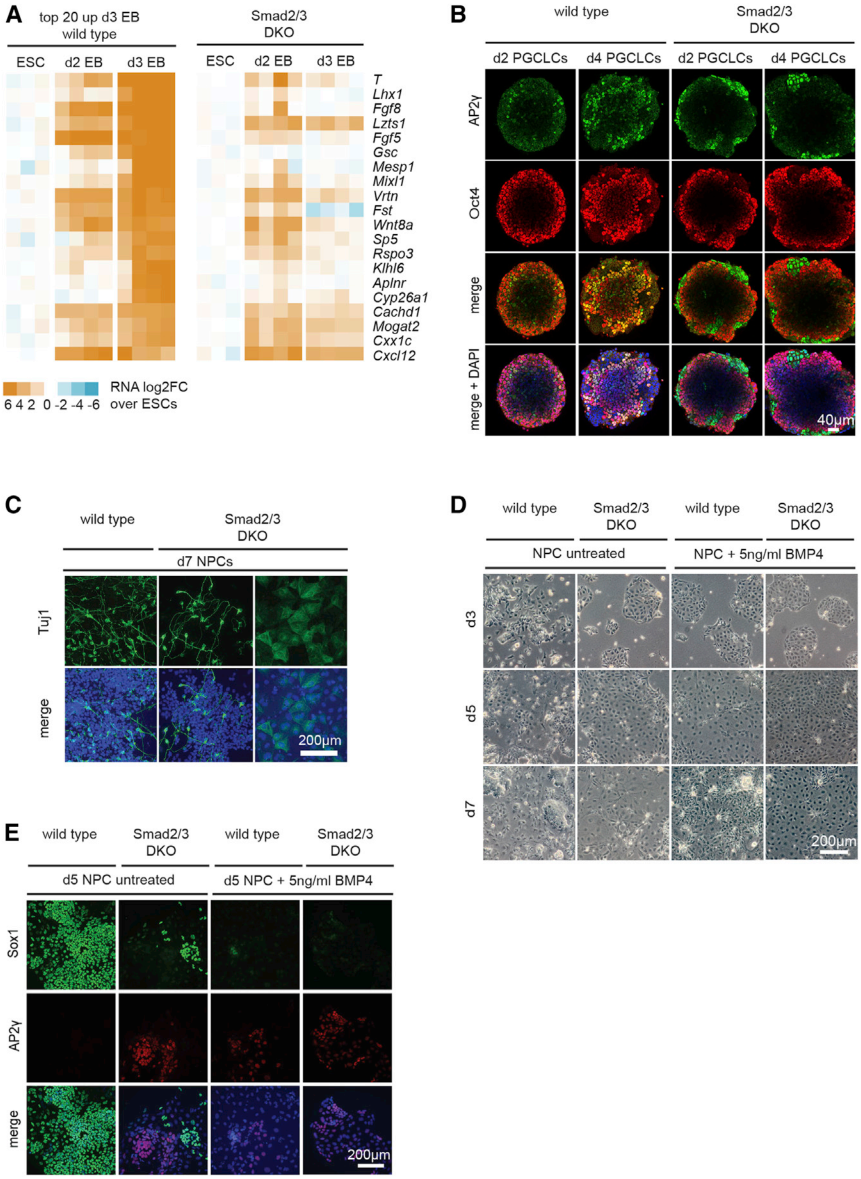

Figure 3. Smad2/3 Governs Embryonic Cell Fate Specification

(A) Heatmap showing the log2 fold change (log2FC) in expression of the top 20 genes upregulated in day 3 WT EBs relative to WT ESCs (left) in comparison with their expression changes in Smad2/3 DKO (right). See also Table S1.

(B) Anti-AP2γ and Oct4 immunofluorescence staining of WT and Smad2/3 DKO day 2 and 4 PGCLCs.

(C) WT and Smad2/3 DKO NPCs at day 7 stained with anti-Tuj1 and counterstained with DAPI.

(D) Bright-field images of control WT or Smad2/3 DKO NPCs grown in the absence or presence of BMP4 (5 ng/mL) at days 3, 5, and 7.

(E) WT and Smad2/3 DKO NPCs grown in the absence or presence of BMP4 (5 ng/mL) stained with anti-Sox1 and Ap2γ and counterstained with DAPI at day 5.

day 3 EBs (1,487 down and 1,617 up) (Figure S3A). Expression of the top 20 genes normally activated during differentiation was dramatically reduced in Smad2/3 DKO EBs (Figure 3A). Expression of several mesodermal (*Wnt3*, *T*, *Fgf8*, *Mixl1*, *Sp8*, *Eomes*, *Mesp1*, and *Lhx1*) and DE lineage marker genes (*Foxa2*, *Sox17*, *Cxcr4*, and *Gata6*), including a subset known to be direct targets of Smad2/3 in EBs (Wang et al., 2017), were significantly downregulated (Table S1). Profiling at an earlier point revealed that expression of a subset of differentiation genes (e.g., *T*, *Cdx2*, *Wnt8a*, and *Sp5*) was initially induced in Smad2/3 DKO day 2 EBs but failed to be maintained. These transcriptional changes were validated for selected genes by immunofluorescent staining (Figure S3B). Experiments analyzing single-KO EBs demonstrate that both Smad2/3 contribute to expression changes (Figures S3A and S3C). When differentially expressed genes in Smad2/3 DKO day 3 EBs were assessed for their relative expression changes in Smad2/3 DKO EpiLCs and ESCs, we found that Smad2/3 is essential for correct gene expression patterns during differentiation at early stages as cells enter the primed state (Figure S3D).

Primordial germ cells (PGCs) are specified in the early epiblast in response to Bmp signaling from the extra-embryonic ectoderm. To test whether PGC specification is affected by loss of Smad2/3, we exploited PGC-like cell (PGCLC) differentiation protocols (Hayashi and Saitou, 2013). AP2γ/Oct4 co-expression identifies PGCs in the early embryo. In WT cultures, Oct4/AP2γ double-positive cells were readily apparent at day 2 and abundant numbers were present at day 4, but by contrast, day 2 and 4 Smad2/3 DKO cultures contained predominantly Oct4 and AP2γ single-positive cells (Figure 3B), allowing us to conclude that their ability to acquire PGC-like fates is also compromised.

Smad2/3 Governs the Balance of Nodal/Bmp Signaling during Ectoderm Specification

To evaluate whether ectodermal cell fate decisions are also Smad2/3 dependent, we exploited culture protocols that promote neuroectodermal precursor cell (NPC) formation. Tuj1+ staining identified a subpopulation of *bona fide* elongated neural cells in day 7 Smad2/3 DKO cultures (Figure 3C). However, as for BMP4-treated WT NPCs (Malaguti et al., 2013), from day 3 onward, most cells displayed a flattened epithelial, surface-ectoderm-like morphology (Figure 3D). At day 5, a high proportion of WT cells expresses the early neural marker Sox1. BMP4 treatment normally represses Sox1 expression in

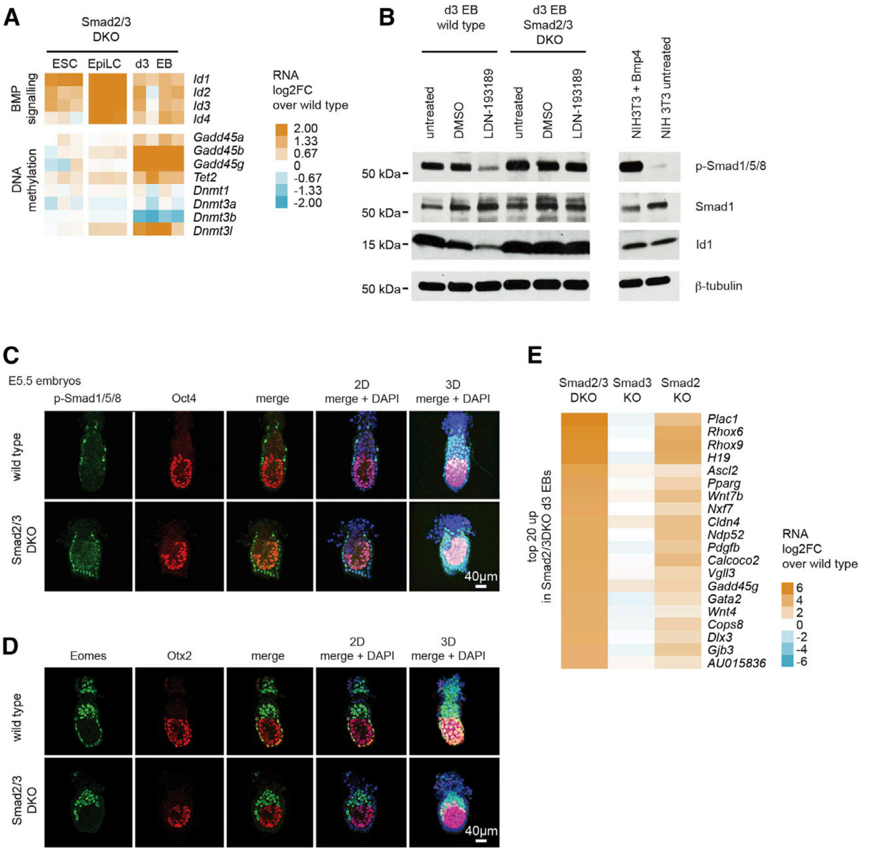

Figure 4. Enhanced Bmp Signaling Caused by the Absence of Smad2/3 Disturbs Embryonic Patterning

(A) Heatmap showing the log2FC in expression of selected Bmp target genes and those involved in DNA methylation for Smad2/3 DKO ESCs, EpiLCs, and day 3 EBs compared to WT controls (n = 3 or 4).

(B) Western blot analysis of WT and Smad2/3 DKO day 3 EBs treated with DMSO or LDN-193189 (250 nM, 24 hr from day 2 to day 3) or untreated. Blots were probed with the indicated antibodies.

(C and D) Anti-p-Smad1/5/8 and Oct4 (C) or Eomes and Otx2 (D) immunofluorescence staining of E5.5 WT or Smad2/3 DKO mouse embryos.

(E) Heatmap showing the log2FC in expression of the top 20 upregulated genes ($p < 0.05$, fold change > 1.5) in Smad2/3 DKO day 3 EBs compared to WT day 3 EBs and their expression changes in Smad2 KO and Smad3 KO day 3 EBs (n = 4, averaged). See also Table S1.

WT cultures (Figures 3E and S3E). In Smad2/3 DKO NPCs, the proportion of Sox1+ cells is markedly reduced, but not eliminated. Similarly, expression of the neural marker *Six3* was absent from BMP4-treated NPCs and significantly reduced in Smad2/3 DKO NPCs (Figure S3E). Moreover, Smad2/3 DKO NPCs exhibited ectopic expression of AP2γ and the epithelial marker *Krt18*, normally present only in BMP4-treated WT cultures (Figures 3E and S3E). Thus, neural fate appears to be induced in a subpopulation of Smad2/3 DKO cells. However, terminal differentiation of neuroectodermal cells is compromised, possibly due to increased Bmp signaling. Thus, as reported for Alk4/5/7 inhibitor-treated ectoderm explants (Li et al., 2013), here we found that combinatorial Smad2/3 activities are required for fine-tuning the balance of neural versus epidermal cell fates.

Ectopic Activation of Extra-Embryonic Gene Expression in Smad2/3-Deficient EBs

Expression of Bmp target genes (*Id1*–*Id4*) was upregulated in Smad2/3 DKO compared to WT ESCs, EpiLCs, and day 3 EBs, consistent with increased levels of Bmp signaling activities (Figure 4A). To examine this possibility, we differentiated Smad2/3 DKO EBs in the presence and absence of the Bmp type 1 receptor inhibitor LDN-193189. In contrast to WT EBs, in which Bmp receptor inhibition markedly reduced p-S1/5/8 levels, we found that Smad2/3 DKO EBs are refractory to LDN-193189 treatment, with levels of p-S1/5/8 and Id1 remaining unchanged (Figure 4B). Next, we evaluated possibly elevated Bmp signaling in embryonic day 5.5 (E5.5) double-mutant embryos. In WT embryos p-S1/5/8 staining is restricted to the proximal VE, while in mutant embryos ectopic nuclear p-S1/5/8 staining is present throughout the VE (Figure 4C). Consistent with this, in double-mutant embryos VE specification is disrupted, as seen by loss of both Eomes and Otx2 expression (Figure 4D).

Bmp signaling has been shown to activate extra-embryonic gene expression (Hayashi et al., 2010). Similarly, in Smad2/3 DKO day 3 EBs, a subset of trophectoderm-derived, extra-embryonic tissue-expressed genes (e.g., *Plac1*, *Rhox6*, *Rhox9*, and *Ascl2*) and VE genes (e.g., *H19*) shows strongly upregulated expression (Figure 4E). However, expression of other essential extra-embryonic genes (e.g., *Elf5*) was unaffected. Thus, we conclude that Smad2/3-deficient cells are not simply defaulting to defined extra-embryonic fates.

Embryonic and extra-embryonic tissues in the early post-implantation mouse embryo show distinct patterns of DNA methylation (Smith et al., 2017). In addition, in the epiblast, DNA methylation gradually increases, coincident with the exit from naive pluripotency (Auclair et al., 2014; Kalkan et al., 2017). Reduced DNA methylation has been shown to cause disturbances, affecting the maintenance of embryonic and/or extra-embryonic cell identity and ESC differentiation (Jackson et al., 2004; Ng et al., 2008; Sakaue et al., 2010). Reduced Bmp signaling in Smad1/5 DKO ESCs results in increased *Dnmt3b* levels, enhanced DNA methylation, and more efficient embryonic differentiation (Gomes Fernandes et al., 2016). Consistent with enhanced Bmp signaling, we found that Smad2/3 DKO day 3 EBs display decreased *Dnmt3b* expression and increased expression of genes associated with DNA demethylation (*Tet2* and *Gadd45b*) (Figure 4A). Extra-embryonically expressed

imprinted genes (e.g., *Rhox5*, *H19*, *Igf2*, *Ascl2*, and *Peg10*), whose differential expression is controlled by DNA methylation, were also upregulated (Table S1). It is tempting to speculate that ectopic extra-embryonic gene expression in Smad2/3-deficient EBs reflects enhanced Bmp signaling, together with changes affecting the patterns of DNA methylation.

DISCUSSION

We reported many years ago that double Smad2/3 homozygous mutant embryos abruptly arrest at early post-implantation stages (Dunn et al., 2004). However, it has proved difficult to characterize the underlying molecular defects responsible for this early lethality. Here we generated double Smad2/3 homozygous null ESCs and investigated their differentiation capabilities. Our genome-wide transcriptional profiling experiments demonstrate that Smad2/3 activities in early epiblast cells are required to promote the transition from naive pluripotency to lineage priming and the onset of cell fate allocation.

Our ATAC-seq analysis of Smad2/3 DKO EpiLCs revealed changes in chromatin accessibility at distal regulatory elements occupied by the pioneer transcription factor Oct4 and its interaction partner Otx2 (Buecker et al., 2014; King and Klose, 2017). These changes were closely associated with expression changes at nearby genes. Oct4 interactions with Smad2/3 were previously described in naive and primed cells (Mullen et al., 2011; Sun et al., 2014). It has been proposed that Smad2/3 facilitates accessibility at Oct4-dependent enhancer sites through interactions with chromatin modifiers and remodelers (Funa et al., 2015; Ruetz et al., 2017). The present experiments support the idea that Smad2/3/Oct4/Otx2 transcription complexes are required to mediate cellular transitions from naive to primed and primed to differentiated states. It will be interesting to learn more about associations with additional cofactors having an impact on chromatin structure and transcriptional output at specific target loci.

Early epiblast cells normally undergo lineage priming in the context of tightly balanced reciprocal Nodal/Smad2 and Bmp/Smad1 signaling cues between the embryonic and the extra-embryonic tissues (Ben-Haim et al., 2006; Yamamoto et al., 2009). The present experiments demonstrate that Smad2/3 inactivation results in upregulated Bmp target and extra-embryonic gene expression. Similarly, in Smad2/3 double-mutant embryos, we find ectopic Bmp signaling throughout the distal VE. Studies also suggest that Bmp signaling promotes DNA hypo-methylation in ESCs (Gomes Fernandes et al., 2016). It is tempting to speculate that Smad2/3 activities normally antagonize Bmp signaling and promote DNA methylation selectively in the early epiblast to maintain its developmental potential and prevent contributions to the extra-embryonic cell lineages.

Smad2/3 DKO embryos are more severely disturbed and die earlier in comparison with Nodal KO embryos (Brennan et al., 2001; Dunn et al., 2004). Similarly, Smad2/3 DKO ESCs display more striking differentiation defects compared with Nodal KO ESCs. For example, Nodal KO embryos and ESCs induced to differentiate have been shown to prematurely activate neural gene expression (Camus et al., 2006; Mulas et al., 2017). In contrast, we found that Smad2/3 DKO ESCs induced to differentiate display reduced neural and enhanced surface ectoderm-like and extra-embryonic gene expression. The simplest explanation is that in the absence of Nodal, closely related Smad2/3-dependent TGF-β family members like Gdf1 and Gdf3 partially compensate (Andersson et al., 2007). Consistent with this possibility, Nodal-deficient blastocysts have the ability to activate the Smad2/3-dependent Nodal anterior streak enhancer (Granier et al., 2011). Moreover, unlike Smad2/3 DKO ESCs, Nodal KO ESCs efficiently contribute to embryonic cell lineages (Conlon et al., 1991). Widespread tissue defects observed in Smad2/3 DKO embryos are also considerably more severe compared with those reported for mutant embryos lacking *Fgf5*, *Oct6*, or *Otx2* (Ang et al., 1996; Bermingham et al., 1996; Hébert et al., 1994). Thus, it appears that the profound developmental block in Smad2/3 DKO embryos reflects not only defective lineage priming and embryonic cell fate allocation but also additional disturbances caused by de-repressed Bmp target gene and ectopic extra-embryonic gene expression. Overall, our experiments demonstrate that combinatorial Smad2/3 functional activities collaboratively maintain distinct embryonic and/or extra-embryonic cell identities and strictly divergent lineage-specific transcriptional programs in the early mouse embryo.

STAR★METHODS

Detailed methods are provided in the online version of this paper and include the following:

- KEY RESOURCES TABLE
- CONTACT FOR REAGENT AND RESOURCE SHARING
- EXPERIMENTAL MODEL AND SUBJECT DETAILS
  - Animal care and use
  - ESC culture
- METHOD DETAILS
  - Generation of knockout lines
  - EpiLC induction
  - PGCLC induction
  - EB differentiation
  - NPC induction
  - ATAC-seq
  - Microarray profiling
  - RNA-seq
  - RT-PCR
  - Immunofluorescence
  - Colony forming assay
  - Alkaline phosphatase staining
- QUANTIFICATION AND STATISTICAL ANALYSIS
  - RT-PCR
  - Microarray analysis
  - ATAC-seq, ChIP-seq and RNA-seq analysis
- DATA AND SOFTWARE AVAILABILITY

SUPPLEMENTAL INFORMATION

Supplemental Information includes three figures and four tables and can be found with this article online at https://doi.org/10.1016/j.celrep.2018.07.077.

ACKNOWLEDGMENTS

We thank Andrew Bassett for his advice with the CRISPR-Cas9n deletion strategy, Thomas Clague and Maria-Eleni Xypolita for technical assistance, and the High-Throughput Genomics Group (Wellcome Trust (WT) Centre for Human Genetics, funded by WT 090532/Z/09/Z), for generating sequencing and microarray data. Confocal microscopy was carried out in the Micron Oxford Advanced Bioimaging Unit (funded by WT Strategic Award 107457). This work was funded by the Wellcome Trust (099840/Z/12/A to A.D.S. and 102811/Z/13/2 to E.J.R.).

AUTHOR CONTRIBUTIONS

A.D.S. and I.C. conducted the experiments. A.D.S., I.C., E.K.B., and E.J.R. designed the experiments. A.D.S. and H.W.K. performed computational analysis of RNA-seq, ChIP-seq, and ATAC-seq data. A.D.S. and A.W.M. analyzed microarray data. A.D.S., I.C., H.W.K., E.K.B., and E.J.R. wrote the paper.

DECLARATION OF INTERESTS

The authors declare no competing interests.

Received: February 20, 2018
Revised: May 31, 2018
Accepted: July 22, 2018
Published: August 21, 2018

REFERENCES

Andersson, O., Bertolino, P., and Ibáñez, C.F. (2007). Distinct and cooperative roles of mammalian Vg1 homologs GDF1 and GDF3 during early embryonic development. Dev. Biol. *311*, 500–511.

Ang, S.L., Jin, O., Rhinn, M., Daigle, N., Stevenson, L., and Rossant, J. (1996). A targeted mouse Otx2 mutation leads to severe defects in gastrulation and formation of axial mesoderm and to deletion of rostral brain. Development *122*, 243–252.

Auclair, G., Guibert, S., Bender, A., and Weber, M. (2014). Ontogeny of CpG island methylation and specificity of DNMT3 methyltransferases during embryonic development in the mouse. Genome Biol. *15*, 545.

Ben-Haim, N., Lu, C., Guzman-Ayala, M., Pescatore, L., Mesnard, D., Bischofberger, M., Naef, F., Robertson, E.J., and Constam, D.B. (2006). The nodal precursor acting via activin receptors induces mesoderm by maintaining a source of its convertases and BMP4. Dev. Cell *11*, 313–323.

Bermingham, J.R., Jr., Scherer, S.S., O'Connell, S., Arroyo, E., Kalla, K.A., Powell, F.L., and Rosenfeld, M.G. (1996). Tst-1/Oct-6/SCIP regulates a unique step in peripheral myelination and is required for normal respiration. Genes Dev. *10*, 1751–1762.

Brennan, J., Lu, C.C., Norris, D.P., Rodriguez, T.A., Beddington, R.S., and Robertson, E.J. (2001). Nodal signalling in the epiblast patterns the early mouse embryo. Nature *411*, 965–969.

Buecker, C., Srinivasan, R., Wu, Z., Calo, E., Acampora, D., Faial, T., Simeone, A., Tan, M., Swigut, T., and Wysocka, J. (2014). Reorganization of enhancer patterns in transition from naive to primed pluripotency. Cell Stem Cell *14*, 838–853.

Buenrostro, J.D., Giresi, P.G., Zaba, L.C., Chang, H.Y., and Greenleaf, W.J. (2013). Transposition of native chromatin for fast and sensitive epigenomic profiling of open chromatin, DNA-binding proteins and nucleosome position. Nat. Methods *10*, 1213–1218.

Camus, A., Perea-Gomez, A., Moreau, A., and Collignon, J. (2006). Absence of Nodal signaling promotes precocious neural differentiation in the mouse embryo. Dev. Biol. *295*, 743–755.

Chen, K., Xi, Y., Pan, X., Li, Z., Kaestner, K., Tyler, J., Dent, S., He, X., and Li, W. (2013). DANPOS: dynamic analysis of nucleosome position and occupancy by sequencing. Genome Res. *23*, 341–351.

Conlon, F.L., Barth, K.S., and Robertson, E.J. (1991). A novel retrovirally induced embryonic lethal mutation in the mouse: assessment of the developmental fate of embryonic stem cells homozygous for the 413.d proviral integration. Development *111*, 969–981.

Datto, M.B., Frederick, J.P., Pan, L., Borton, A.J., Zhuang, Y., and Wang, X.F. (1999). Targeted disruption of Smad3 reveals an essential role in transforming growth factor beta-mediated signal transduction. Mol. Cell. Biol. *19*, 2495–2504.

Dobin, A., Davis, C.A., Schlesinger, F., Drenkow, J., Zaleski, C., Jha, S., Batut, P., Chaisson, M., and Gingeras, T.R. (2013). STAR: ultrafast universal RNA-seq aligner. Bioinformatics *29*, 15–21.

Dunn, N.R., Vincent, S.D., Oxburgh, L., Robertson, E.J., and Bikoff, E.K. (2004). Combinatorial activities of Smad2 and Smad3 regulate mesoderm formation and patterning in the mouse embryo. Development *131*, 1717–1728.

Dunn, N.R., Koonce, C.H., Anderson, D.C., Islam, A., Bikoff, E.K., and Robertson, E.J. (2005). Mice exclusively expressing the short isoform of Smad2 develop normally and are viable and fertile. Genes Dev. *19*, 152–163.

Eijssen, L.M., Goelela, V.S., Kelder, T., Adriaens, M.E., Evelo, C.T., and Radonjic, M. (2015). A user-friendly workflow for analysis of Illumina gene expression bead array data available at the arrayanalysis.org portal. BMC Genomics *16*, 482.

Factor, D.C., Corradin, O., Zentner, G.E., Saiakhova, A., Song, L., Chenoweth, J.G., McKay, R.D., Crawford, G.E., Scacheri, P.C., and Tesar, P.J. (2014). Epigenomic comparison reveals activation of "seed" enhancers during transition from naive to primed pluripotency. Cell Stem Cell *14*, 854–863.

Funa, N.S., Schachter, K.A., Lerdrup, M., Ekberg, J., Hess, K., Dietrich, N., Honoré, C., Hansen, K., and Semb, H. (2015). β-catenin regulates primitive streak induction through collaborative interactions with SMAD2/SMAD3 and OCT4. Cell Stem Cell *16*, 639–652.

Gomes Fernandes, M., Dries, R., Roost, M.S., Semrau, S., de Melo Bernardo, A., Davis, R.P., Ramakrishnan, R., Szuhai, K., Maas, E., Umans, L., et al. (2016). BMP-SMAD signaling regulates lineage priming, but is dispensable for self-renewal in mouse embryonic stem cells. Stem Cell Reports *6*, 85–94.

Granier, C., Gurchenkov, V., Perea-Gomez, A., Camus, A., Ott, S., Papanayotou, C., Iranzo, J., Moreau, A., Reid, J., Koentges, G., et al. (2011). Nodal *cis*-regulatory elements reveal epiblast and primitive endoderm heterogeneity in the peri-implantation mouse embryo. Dev. Biol. *349*, 350–362.

Hayashi, K., and Saitou, M. (2013). Generation of eggs from mouse embryonic stem cells and induced pluripotent stem cells. Nat. Protoc. *8*, 1513–1524.

Hayashi, Y., Furue, M.K., Tanaka, S., Hirose, M., Wakisaka, N., Danno, H., Ohnuma, K., Oeda, S., Aihara, Y., Shiota, K., et al. (2010). BMP4 induction of trophoblast from mouse embryonic stem cells in defined culture conditions on laminin. In Vitro Cell. Dev. Biol. Anim. *46*, 416–430.

Hayashi, K., Ohta, H., Kurimoto, K., Aramaki, S., and Saitou, M. (2011). Reconstitution of the mouse germ cell specification pathway in culture by pluripotent stem cells. Cell *146*, 519–532.

Hébert, J.M., Rosenquist, T., Götz, J., and Martin, G.R. (1994). FGF5 as a regulator of the hair growth cycle: evidence from targeted and spontaneous mutations. Cell *78*, 1017–1025.

Heinz, S., Benner, C., Spann, N., Bertolino, E., Lin, Y.C., Laslo, P., Cheng, J.X., Murre, C., Singh, H., and Glass, C.K. (2010). Simple combinations of lineage-determining transcription factors prime *cis*-regulatory elements required for macrophage and B cell identities. Mol. Cell *38*, 576–589.

Hsu, P.D., Scott, D.A., Weinstein, J.A., Ran, F.A., Konermann, S., Agarwala, V., Li, Y., Fine, E.J., Wu, X., Shalem, O., et al. (2013). DNA targeting specificity of RNA-guided Cas9 nucleases. Nat. Biotechnol. *31*, 827–832.

Hulsen, T., de Vlieg, J., and Alkema, W. (2008). BioVenn—a web application for the comparison and visualization of biological lists using area-proportional Venn diagrams. BMC Genomics *9*, 488.

Jackson, M., Krassowska, A., Gilbert, N., Chevassut, T., Forrester, L., Ansell, J., and Ramsahoye, B. (2004). Severe global DNA hypomethylation blocks differentiation and induces histone hyperacetylation in embryonic stem cells. Mol. Cell. Biol. *24*, 8862–8871.

Kalkan, T., Olova, N., Roode, M., Mulas, C., Lee, H.J., Nett, I., Marks, H., Walker, R., Stunnenberg, H.G., Lilley, K.S., et al. (2017). Tracking the embryonic stem cell transition from ground state pluripotency. Development *144*, 1221–1234.

King, H.W., and Klose, R.J. (2017). The pioneer factor OCT4 requires the chromatin remodeller BRG1 to support gene regulatory element function in mouse embryonic stem cells. eLife *6*, e22631.

Langmead, B., and Salzberg, S.L. (2012). Fast gapped-read alignment with Bowtie 2. Nat. Methods *9*, 357–359.

Li, H., Handsaker, B., Wysoker, A., Fennell, T., Ruan, J., Homer, N., Marth, G., Abecasis, G., and Durbin, R.; 1000 Genome Project Data Processing Subgroup (2009). The Sequence Alignment/Map format and SAMtools. Bioinformatics *25*, 2078–2079.

Li, L., Liu, C., Biechele, S., Zhu, Q., Song, L., Lanner, F., Jing, N., and Rossant, J. (2013). Location of transient ectodermal progenitor potential in mouse development. Development *140*, 4533–4543.

Love, M.I., Huber, W., and Anders, S. (2014). Moderated estimation of fold change and dispersion for RNA-seq data with DESeq2. Genome Biol. *15*, 550.

Malaguti, M., Nistor, P.A., Blin, G., Pegg, A., Zhou, X., and Lowell, S. (2013). Bone morphogenic protein signalling suppresses differentiation of pluripotent cells by maintaining expression of E-cadherin. eLife *2*, e01197.

Massagué, J. (2012). TGFβ signalling in context. Nat. Rev. Mol. Cell Biol. *13*, 616–630.

McLean, C.Y., Bristor, D., Hiller, M., Clarke, S.L., Schaar, B.T., Lowe, C.B., Wenger, A.M., and Bejerano, G. (2010). GREAT improves functional interpretation of *cis*-regulatory regions. Nat. Biotechnol. *28*, 495–501.

McLeay, R.C., and Bailey, T.L. (2010). Motif enrichment analysis: a unified framework and an evaluation on ChIP data. BMC Bioinformatics *11*, 165.

Mesnard, D., Guzman-Ayala, M., and Constam, D.B. (2006). Nodal specifies embryonic visceral endoderm and sustains pluripotent cells in the epiblast before overt axial patterning. Development *133*, 2497–2505.

Morgani, S., Nichols, J., and Hadjantonakis, A.K. (2017). The many faces of pluripotency: *in vitro* adaptations of a continuum of *in vivo* states. BMC Dev. Biol. *17*, 7.

Morikawa, M., Koinuma, D., Mizutani, A., Kawasaki, N., Holmborn, K., Sundqvist, A., Tsutsumi, S., Watabe, T., Aburatani, H., Heldin, C.H., and Miyazono, K. (2016). BMP sustains embryonic stem cell self-renewal through distinct functions of different Krüppel-like factors. Stem Cell Reports *6*, 64–73.

Mulas, C., Kalkan, T., and Smith, A. (2017). NODAL secures pluripotency upon embryonic stem cell progression from the ground state. Stem Cell Reports *9*, 77–91.

Mullen, A.C., Orlando, D.A., Newman, J.J., Lovén, J., Kumar, R.M., Bilodeau, S., Reddy, J., Guenther, M.G., DeKoter, R.P., and Young, R.A. (2011). Master transcription factors determine cell-type-specific responses to TGF-β signaling. Cell *147*, 565–576.

Murakami, K., Günesdogan, U., Zylicz, J.J., Tang, W.W.C., Sengupta, R., Kobayashi, T., Kim, S., Butler, R., Dietmann, S., and Surani, M.A. (2016). NANOG alone induces germ cells in primed epiblast *in vitro* by activation of enhancers. Nature *529*, 403–407.

Ng, R.K., Dean, W., Dawson, C., Lucifero, D., Madeja, Z., Reik, W., and Hemberger, M. (2008). Epigenetic restriction of embryonic cell lineage fate by methylation of Elf5. Nat. Cell Biol. *10*, 1280–1290.

Quinlan, A.R., and Hall, I.M. (2010). BEDTools: a flexible suite of utilities for comparing genomic features. Bioinformatics *26*, 841–842.

Ran, F.A., Hsu, P.D., Lin, C.Y., Gootenberg, J.S., Konermann, S., Trevino, A.E., Scott, D.A., Inoue, A., Matoba, S., Zhang, Y., and Zhang, F. (2013). Double nicking by RNA-guided CRISPR Cas9 for enhanced genome editing specificity. Cell *154*, 1380–1389.

Robertson, E.J. (2014). Dose-dependent Nodal/Smad signals pattern the early mouse embryo. Semin. Cell Dev. Biol. *32*, 73–79.

Robertson, E., Bradley, A., Kuehn, M., and Evans, M. (1986). Germ-line transmission of genes introduced into cultured pluripotential cells by retroviral vector. Nature *323*, 445–448.

Ross-Innes, C.S., Stark, R., Teschendorff, A.E., Holmes, K.A., Ali, H.R., Dunning, M.J., Brown, G.D., Gojis, O., Ellis, I.O., Green, A.R., et al. (2012). Differential oestrogen receptor binding is associated with clinical outcome in breast cancer. Nature *481*, 389–393.

Ruetz, T., Pfisterer, U., Di Stefano, B., Ashmore, J., Beniazza, M., Tian, T.V., Kaemena, D.F., Tosti, L., Tan, W., Manning, J.R., et al. (2017). Constitutively active SMAD2/3 are broad-scope potentiators of transcription-factor-mediated cellular reprogramming. Cell Stem Cell *21*, 791–805.e9.

Sakaue, M., Ohta, H., Kumaki, Y., Oda, M., Sakaide, Y., Matsuoka, C., Yamagiwa, A., Niwa, H., Wakayama, T., and Okano, M. (2010). DNA methylation is dispensable for the growth and survival of the extraembryonic lineages. Curr. Biol. *20*, 1452–1457.

Saldanha, A.J. (2004). Java Treeview—extensible visualization of microarray data. Bioinformatics *20*, 3246–3248.

Schindelin, J., Arganda-Carreras, I., Frise, E., Kaynig, V., Longair, M., Pietzsch, T., Preibisch, S., Rueden, C., Saalfeld, S., Schmid, B., et al. (2012). Fiji: an open-source platform for biological-image analysis. Nat. Methods *9*, 676–682.

Simon, C.S., Downes, D.J., Gosden, M.E., Telenius, J., Higgs, D.R., Hughes, J.R., Costello, I., Bikoff, E.K., and Robertson, E.J. (2017). Functional characterisation of *cis*-regulatory elements governing dynamic *Eomes* expression in the early mouse embryo. Development *144*, 1249–1260.

Smith, A. (2017). Formative pluripotency: the executive phase in a developmental continuum. Development *144*, 365–373.

Smith, Z.D., Shi, J., Gu, H., Donaghey, J., Clement, K., Cacchiarelli, D., Gnirke, A., Michor, F., and Meissner, A. (2017). Epigenetic restriction of extraembryonic lineages mirrors the somatic transition to cancer. Nature *549*, 543–547.

Stark, R. and Brown, G., eds. (2011). DiffBind: Differential Binding Analysis of ChIP-Seq Peak Data (Bioconductor).

Sun, L.T., Yamaguchi, S., Hirano, K., Ichisaka, T., Kuroda, T., and Tada, T. (2014). Nanog co-regulated by Nodal/Smad2 and Oct4 is required for pluripotency in developing mouse epiblast. Dev. Biol. *392*, 182–192.

Tremblay, K.D., Hoodless, P.A., Bikoff, E.K., and Robertson, E.J. (2000). Formation of the definitive endoderm in mouse is a Smad2-dependent process. Development *127*, 3079–3090.

Vincent, S.D., Dunn, N.R., Hayashi, S., Norris, D.P., and Robertson, E.J. (2003). Cell fate decisions within the mouse organizer are governed by graded Nodal signals. Genes Dev. *17*, 1646–1662.

Waldrip, W.R., Bikoff, E.K., Hoodless, P.A., Wrana, J.L., and Robertson, E.J. (1998). Smad2 signaling in extraembryonic tissues determines anterior-posterior polarity of the early mouse embryo. Cell *92*, 797–808.

Wang, Q., Zou, Y., Nowotschin, S., Kim, S.Y., Li, Q.V., Soh, C.L., Su, J., Zhang, C., Shu, W., Xi, Q., et al. (2017). The p53 family coordinates Wnt and Nodal inputs in mesendodermal differentiation of embryonic stem cells. Cell Stem Cell *20*, 70–86.

Yamamoto, M., Beppu, H., Takaoka, K., Meno, C., Li, E., Miyazono, K., and Hamada, H. (2009). Antagonism between Smad1 and Smad2 signaling determines the site of distal visceral endoderm formation in the mouse embryo. J. Cell Biol. *184*, 323–334.

Ying, Q.L., Wray, J., Nichols, J., Batlle-Morera, L., Doble, B., Woodgett, J., Cohen, P., and Smith, A. (2008). The ground state of embryonic stem cell self-renewal. Nature *453*, 519–523.

Zhang, Y., Liu, T., Meyer, C.A., Eeckhoute, J., Johnson, D.S., Bernstein, B.E., Nusbaum, C., Myers, R.M., Brown, M., Li, W., and Liu, X.S. (2008). Model-based analysis of ChIP-seq (MACS). Genome Biol. *9*, R137.

STAR★METHODS

KEY RESOURCES TABLE

REAGENT or RESOURCE	SOURCE	IDENTIFIER
Antibodies		
Rabbit polyclonal anti-mouse Nanog	Abcam	Cat#ab80892; RRID: AB_2150114 Lot: GR40243-12
Goat polyclonal anti-mouse Oct4	Santa Cruz	Cat#sc-8628; RRID: AB_653551, Lot: F1815
Mouse monoclonal anti-mouse Klf4	Santa Cruz	Cat#sc-393462, Lot: iO116
Rabbit polyclonal anti-human Ap2γ	Santa Cruz	Cat#sc-8977; RRID: AB_2286995, Lot: G1112
Goat polyclonal anti-human Brachyury (N-19)	Santa Cruz	Cat#sc-17743; RRID: AB_634980, Lot: A1614
Rat monoclonal anti-mouse E-Cadherin	Sigma-Aldrich	Cat#U3254; RRID: AB_477600, Lot: 085K4798
Goat polyclonal anti-human Gata6	R&D Systems	Cat#AF1700; RRID: AB_2108901, Lot: KWT-417101
Mouse monoclonal anti-human Cdx2	BioGenex	Cat#MU392A-UC; RRID: AB_2650531, Lot: MU392A0713
Rabbit polyclonal anti-mouse Eomes	Abcam	Cat#ab23345; RRID: AB_778267, Lot:GR306193-1
Rabbit monoclonal anti-human Smad1/5/8 (embryo)	Cell Signaling Technology	Cat#13820; RRID: AB_2493181, Lot: D5810
Rabbit polyclonal anti-human Smad1/5/8 (Western blot)	Merck-Millipore	Cat#AB3848; RRID: AB_628261, Lot: JBC17774748
Mouse monoclonal anti-human Smad1	Santa Cruz	Cat#sc-7965; RRID: AB_628261, Lot: A072
Rabbit polyclonal anti-mouse Id1	Santa Cruz	Cat#sc-488; RRID: AB_631701, Lot: B082
Mouse monoclonal anti-mouse p-Smad2	BD Transduction	Cat#610843; RRID:AB_398162, Lot: 3288899
Rabbit monoclonal anti-human Smad3	Abcam	Cat#ab40854; RRID: AB_777979, Lot: GR16548-6
Rabbit polyclonal anti-human Tubulin	Santa Cruz	Cat#sc-9104; RRID: AB_2241191, Lot:I1009
Mouse polyclonal anti-rat Tuj1	Bio Legend	Cat#801201; RRID: AB_2313773, Lot: B209227
Goat polyclonal anti-human Sox1	R&D Systems	Cat#AF3369; RRID: AB_2239879, Lot:XUV0417031
Goat polyclonal anti-human Otx2	R&D Systems	Cat#AF1979; RRID: AB_2157172, Lot: KNO0615111
Goat polyclonal anti-human Oct6	Santa Cruz	Cat#sc-11661; RRID: AB_2268536
Donkey anti-goat Alexa 594	Molecular Probes	Cat#A11058; RRID: AB_142540
Donkey anti-goat Alexa 488	Molecular Probes	Cat#A11055; RRID: AB_142672
Donkey anti-mouse Alexa 488	Molecular Probes	Cat#A21202; RRID: AB_141607
Donkey anti-rabbit Alexa 594	Thermo Fisher Scientific	Cat#A21207; RRID: AB_141637
Donkey anti-goat Alexa 594	Molecular Probes	Cat#A11058; RRID: AB_142540
Donkey anti-rabbit Alexa 488	Molecular Probes	Cat#A21206; RRID: AB_141708
Donkey anti-rat 594	Molecular Probes	Cat#A21209; RRID: AB_2535795
Donkey anti-rabbit HRP	GE Healthcare	Cat#NA934; RRID: AB_772206
Sheep anti-mouse HRP	GE Healthcare	Cat#NA931; RRID: AB_772212

(*Continued on next page*)

Continued

REAGENT or RESOURCE	SOURCE	IDENTIFIER
Bacterial and Virus Strains		
One Shot Stbl3 Chemically Competent *E. coli*	Invitrogen	Cat#C737303
Chemicals, Peptides, and Recombinant Proteins		
Recombinant human/murine/rat Activin A	Peprotech	Cat#120-14E, Lot: 1115478-1
Recombinant human bFGF	Invitrogen	Cat#13256-029, Lot: 1711319A
LDN-193189 (small molecule inhibitor)	Stemgent	Cat#04-0074, Lot: 3061
Recombinant human BMP4	R&D Systems	Cat#314-BP, Lot: BEM11816121
Recombinant mouse SCF	R&D Systems	Cat#455-MC, Lot: CW1715062
Recombinant human Bmp8	R&D Systems	Cat#1073-BP, Lot: EXU1116031
Recombinant mouse EGF	R&D Systems	Cat#2028-EG, Lot: MKG1016021
Human plasma fibronectin purified protein	Millipore	Cat#FC010
CHIR99021	Synthesized by the MRC Protein Phosphorylation Unit, Division of Signal Transduction Therapy at the University of Dundee, UK	N/A
PD0325091	Synthesized by the MRC Protein Phosphorylation Unit, Division of Signal Transduction Therapy at the University of Dundee, UK	N/A
Recombinant LIF (ESGRO)	Millipore	Cat#ESG1107, Lot: 2710245
Critical Commercial Assays		
Alkaline Phosphatase Detection Kit	Millipore	Cat#SCR004
Nextera DNA Library Preparation Kit	Illumina	Cat#FC-121-1030
Deposited Data		
RNA-seq on Smad2/3 DKO and control epiblast-like cells	N/A	GEO: GSE110163, Table S2, Series GSE110164
ATAC-seq on Smad2/3 DKO and control epiblast-like cells (including Tn5 control)	N/A	GEO: GSE110162, Table S3, Series GSE110164
Illumina MouseWG-6 v2.0 Expression BeadChip microarray on Smad2/3 DKO, Smad2 KO, Smad3 KO and WT mouse ESCs, d2 and d3 EBs	N/A	GEO: GSE110058, Table S1, Series GSE110164
Experimental Models: Cell Lines		
WT CCE 129/Sv//Ev embryonic stem cells	(Robertson et al., 1986)	N/A
Smad2$^{tm1Rob/tm1Rob}$ 129/Sv//Ev embryonic stem cells	(Tremblay et al., 2000)	N/A
Smad3$^{tm1Xfw/tm1Xfw}$ 129/Sv * C57BL/6 embryonic stem cells	This study	N/A
Smad3$^{CRISPR/CRISPR}$ 129/Sv//Ev embryonic stem cells	This study	N/A
Smad2$^{tm1Rob/tm1Rob}$;*Smad3*$^{CRISPR/CRISPR}$ 129/Sv//Ev embryonic stem cells	This study	N/A
NIH 3T3 cells	ATCC	RRID: CVCL_0594
Experimental Models: Organisms/Strains		
Mouse: *Smad3*^{tm1Xfw}/+: C57BL/6	Obtained from (Datto et al., 1999)	RRID: MGI:2182651
Mouse: Smad2^{tm1Rob}/+: C57BL/6	(Waldrip et al., 1998)	MGI:1857691
Oligonucleotides		
Smad3 CRISPR_5′ nick 1 forward CACCGCCCACG TGGGCCACCGGGTAGGG	This study	N/A
Smad3 CRISPR_5′ nick 1 reverse AAACTACCCGGT GGCCCACGTGGGC	This study	N/A

(*Continued on next page*)

Continued

REAGENT or RESOURCE	SOURCE	IDENTIFIER
Smad3 CRISPR_5′ nick 2 forward CACCGCGCTG GCGGCGCTGGGCGGGG	This study	N/A
Smad3 CRISPR_5′ nick 2 reverse AAACCGCCCA GCGCCGCCAAGCGC	This study	N/A
Smad3 CRISPR_3′ nick 1 forward CACCGTGTCCC GCCCCACTCGAAGCGC	This study	N/A
Smad3 CRISPR_3′ nick 1 reverse AAACCCGCTT CGAGTGGGGCGGGACAC	This study	N/A
Smad3 CRISPR_3′ nick 2 forward CACCGTCAGTA CATTCTGTCAGATCTGG	This study	N/A
Smad3 CRISPR_3′ nick 2 reverse AAACCCAGAT CTGACAGAATGTACTGAC	This study	N/A
CRISPR_U6 sequencing primer forward GACTATCAT ATGCTTACCGT	This study	N/A
Primers for qRT-PCR and OneStep RT-PCR analysis as well as mouse genotyping and Southern blot probe, see Table S4	This study	N/A
Recombinant DNA		
pSpCas9n(BB)-2A-GFP (PX461)	(Ran et al., 2013)	Addgene Plamid #18140
Software and Algorithms		
CRISPR design tool	(Hsu et al., 2013)	http://crispr.mit.edu
Bowtie2 aligner	(Langmead and Salzberg, 2012)	http://bowtie-bio.sourceforge.net/bowtie2/index.shtml
STAR aligner	(Dobin et al., 2013)	https://github.com/alexdobin/STAR
Samtools	(Li et al., 2009)	http://samtools.sourceforge.net/
Danpos2	(Chen et al., 2013)	https://sites.google.com/site/danposdoc/
MACS2	(Zhang et al., 2008)	https://github.com/taoliu/MACS
bedtools	(Quinlan and Hall, 2010)	http://bedtools.readthedocs.io/en/latest/#
UCSC Genome Browser		https://genome.ucsc.edu/
DiffBind R package	(Ross-Innes et al., 2012)	https://bioconductor.org/packages/release/bioc/html/DiffBind.html
DESeq2 R package	(Love et al., 2014)	http://bioconductor.org/packages/release/bioc/html/DESeq2.html
HOMER	(Heinz et al., 2010)	http://homer.ucsd.edu/homer/index.html
JavaTreeView	(Saldanha, 2004)	http://jtreeview.sourceforge.net/
Analysis of Motif Enrichment Feature in the MEME suite	(McLeay and Bailey, 2010)	http://meme-suite.org/doc/ame.html?man_type=web
GREAT	(McLean et al., 2010)	http://bejerano.stanford.edu/great/public/html/
Arrayanalysis	(Eijssen et al., 2015)	http://www.arrayanalysis.org/
BioVenn	(Hulsen et al., 2008)	http://www.biovenn.nl/
Fiji	(Schindelin et al., 2012)	https://imagej.net/Fiji
Other		
Mus musculus rRNA genomic sequence	GenBank	BK000964.3
mm10 genome	UCSC Genome Browser	http://hgdownload.cse.ucsc.edu/goldenPath/mm10/bigZips/
Sequencing data from Oct4, Otx2, p300, H3K4me1 and H3K27ac ChIP-seq in EpiLCs and RNA-seq in mouse ESCs and EpiLCs	(Buecker et al., 2014)	GSE56138

(*Continued on next page*)

Continued

REAGENT or RESOURCE	SOURCE	IDENTIFIER
Sequencing data from Smad2/3 ChIP-seq in mouse ESCs and embryoid bodies treated with Activin A or SB-431242	(Wang et al., 2017)	GSE70486
Sequencing data from ATAC-seq in mouse ESCs and *in vitro* differentiated definitive endoderm	(Simon et al., 2017)	GSE94250
Sequencing data from Smad1 ChIP-seq in mouse ESCs treated with BMP4	(Morikawa et al., 2016)	GSE70581

CONTACT FOR REAGENT AND RESOURCE SHARING

Further information and requests for resources and reagents should be directed to and will be fulfilled by the Lead Contact, Elizabeth Robertson (elizabeth.robertson@path.ox.ac.uk).

EXPERIMENTAL MODEL AND SUBJECT DETAILS

Animal care and use

E5.5 Smad2/3 DKO embryos were obtained from intercrosses of *Smad3*$^{tm1Xfw/+}$;*Smad2*$^{tm1Rob/+}$ (Waldrip et al., 1998; Datto et al., 1999) animals. Blastocysts for ESC derivation and thymus tissue for protein lysates were obtained from the *Smad3*^{tm1Xfw} C57BL/6 mouse strain (Datto et al., 1999). PCR genotyping primers are listed in Table S4. All animal experiments were performed in accordance with Home Office (UK) regulations and approved by the University of Oxford Local Ethical Committee.

ESC culture

All ESC lines used were XY and grown in feeder-free conditions on 0.1% gelatin-coated dishes at 6% CO_2 at 37°C. ESCs were cultured in DMEM (GIBCO, Cat#11960-085) supplemented with 15% FBS (GIBCO Cat#10500-062, Lot: 07Q3446K), 1% PEN/STREP, 1% glutamine, 1% NEAA, 1mM sodium pyruvate, 100 μM β-mercaptoethanol and 1000 U/ml LIF (SL). Alternatively, ESCs were cultured in serum-free media containing N2B27 (NDiff®227, Cat#Y40002) supplemented with 1 μM PD0325091 and 3 μM CHIR99021 and 1000 U/ml LIF (2iL).

METHOD DETAILS

Generation of knockout lines

Smad3 KO and Smad2/3 DKO ESC lines

Two sets of four sgRNAs flanking exon 1 of murine *Smad3* were designed using the Zhang lab CRISPR design tool (Hsu et al., 2013) taking care to avoid T-rich 3′ ends and to keep off-sets between nickase-sgRNAs < 10 bp. The PAM sequence was removed and *BbsI* sites engineered at the ends. After cloning into pSpCas9n(BB)-2A-GFP (PX461) (Ran et al., 2013) constructs were transfected into Stbl3 cells. Efficiency of sgRNA plasmids was confirmed by high resolution melt analysis. A maximum of 5 μg pooled isolated non-linearized plasmid DNAs (QIAGEN Maxi Prep kit, Cat#12663) was transfected into 1 × 10^6 ESCs (either 129/Sv//Ev CCE WT (Robertson et al., 1986) or *Smad2*$^{tm1Rob/tm1Rob}$ (Tremblay et al., 2000)) using the Neon® transfection system (Thermo Fisher Scientific, Cat#MPK5000) followed by low-density plating. Deletion of exon 1 was screened for by Southern blotting using a *XbaI* digest and a probe 3′ to the deletion (for sequences see Table S4). Loss of Smad3 protein was confirmed by western blotting using specific antibodies (see Key Resources Table).

Conventional Smad3$^{-/-}$ *control lines*

To generate *Smad3*$^{-/-}$ ESCs, blastocysts were obtained from *Smad3*$^{tm1Xfw/+}$ females mated to *Smad3*$^{tm1Xfw/+}$ males. ESC lines were isolated in 2iL as previously described (Ying et al., 2008). Homozygous lines were identified by PCR using the genotyping primers listed in Table S4.

EpiLC induction

EpiLCs were induced from ESCs (2iL) as previously described (Hayashi et al., 2011). In brief, 2.33 × 10^5 cells were washed and resuspended in N2B27 medium (Takara, Cat#Y40002) supplemented with 12ng/ml Fgf2, 20ng/ml Activin A and 1% KSR (GIBCO, Cat#10828, Lot:1508151) and grown on fibronectin-coated (5 μg/cm^2) 6cm dishes. Medium was exchanged daily and cells grown for 48h to form EpiLCs.

PGCLC induction

PGCLCs were induced from d2 EpiLCs as described previously (Hayashi and Saitou, 2013). In brief, 2000 cells were washed and plated into lipidure-coated U-bottom shaped 96-well plates in serum-free medium (GK15; GMEM (Invitrogen) with 15% KSR, 0.1 mM NEAA, 1 mM sodium pyruvate, 0.1 mM 2-mercaptoethanol, 100 U/ml penicillin, 0.1 mg/ml streptomycin, and 2 mM L-glutamine) in the presence of the cytokines BMP4 (500 ng/ml; R&D Systems), LIF (1000 u/ml; Invitrogen), SCF (100 ng/ml; R&D Systems), BMP8b (500 ng/ml; R&D Systems), and EGF (50 ng/ml; R&D Systems).

EB differentiation

ESCs (SL) were trypsinized, resuspended in serum-containing medium without LIF (EB medium), pelleted, washed in PBS (w/o $MgCl_2$) and counted. A single-cell solution of 1×10^4 cells/ml in EB medium was pipetted into 10 μL drops onto square 12cm plates and inverted to generate hanging drops. After 48 h the resultant EBs were harvested and either collected (d2 EB) or kept in suspension for further 24h (d3 EB) or 48h (d4 EB). For LDN-193189 treatment, d2 EBs were harvested as above after 48h and plated in EB medium containing LDN-193189 (250nM; Stemgent) or equal amounts of DMSO carrier for 24h. As controls, serum-starved NIH 3T3 cells were treated with Bmp4 (50ng/ml) for 30min (or left untreated).

NPC induction

ESCs (SL) were trypsinized when 70%–80% confluent and washed twice in N2B27. 1×10^4 cells/cm^2 were plated in N2B27 medium on fibronectin-coated (5 μg/cm^2) dishes. Medium was changed on the second day of differentiation and then daily.

ATAC-seq

Tagmentation and indexing of single cell suspensions (75,000 cells in technical duplicates) of three independent differentiation of Smad2/3 DKO or WT EpiLCs was performed as previously described (Buenrostro et al., 2013). To control for sequence bias of the Tn5 transposase, 100ng genomic DNA of WT EpiLCs was also tagmented and indexed. Samples were sequenced using a 75-cycle paired end Nextera kit with custom Nextera index primers Ad2.1-2.13 taken from Table S1 in Buenrostro et al. (2013) on the Illumina HiSeq4000 platform.

Microarray profiling

d2 and d3 EBs were harvested, washed and total RNA isolated (QIAGEN RNAeasy micro kit, Cat# 74004). Four independent clones were used per genotype. Undifferentiated cells were collected prior to EB set-up. Biotinylated cRNA (1.5 μg RNA per sample) was randomly hybridized to Illumina MouseWG-6 v2.0 Expression BeadChip microarrays.

RNA-seq

RNA was isolated from ~1.5 x10^6 Smad2/3 DKO and WT EpiLCs from three independent EpiLC differentiations per genotype, using samples taken from the same cells used for ATAC-seq (QIAGEN RNeasy mini kit, Cat#74104). Total RNA was normalized to 800ng per sample, depleted of cytoplasmic and mitochondrial ribosomal RNA sequences (Ribo-Zero Gold rRNA Removal Kit (H/M/R), Cat: #MRZG12324) and used for library preparation using the Illumina TruSeq Stranded Total RNA Library Prep (H/M/R) (Cat: #20020597), followed by sequencing (75-cycle paired end) on the Illumina HiSeq4000 platform.

RT-PCR

1 μg RNA was reverse transcribed to cDNA using Superscript III First Strand Synthesis System (Life Technologies, Cat#18080-051) and diluted to 160 μL final volume in H_2O. 2 μL were used per qRT-PCR reaction in duplicate using SYBR-green kit (QIAGEN, Cat#204143). Relative gene expression was normalized to *Gapdh* expression and calculated as $2^{\Delta\Delta}$Ct. OneStep RT-PCR analysis was performed on 50ng RNA using OneStep RT-PCR kit (QIAGEN, Cat#210210) following the manufacturers protocols. Samples were run on 2% agarose/1xTAE gels. qRT-PCR and OneStep RT-PCR primer sequences are listed in Table S4.

Immunofluorescence

ESCs or EpiLCs grown in 8-well chamber slides were washed twice in PBS (with $MgCl_2$) and fixed in 4% PFA (10min at RT). After three further washes, cells were permeabilized in PBS plus 0.2% Triton X-100, followed by two washes in PBS plus 0.05% Tween-20 (PBST) then blocked (10% donkey serum and 1% BSA in PBST for 1h at RT) and incubated with primary antibodies in blocking solution (see above) (o/n at 4°C). Following two washes in PBST, cells were incubated with fluorescence-labeled secondary antibodies in blocking solution for 1h at RT followed by two washes in PBST containing 2 μg/ml DAPI prior to mounting in Vectashield with DAPI (H-1200) and imaging on a Leica epifluorescence microscope. d2 and d3 EBs, d2 and d4 PGCLCs and E5.5 mouse embryos were harvested, washed in PBS (with $MgCl_2$), fixed in 1% PFA o/n at 4°C. After three washes in PBS containing 0.1% Triton X-100 (PBSTr), samples were permeabilized in PBS containing 0.5% Triton X-100 followed by three washes in PBSTr then blocked (5% donkey serum and 0.2% BSA in PBSTr for 1h at RT) and incubated with primary antibodies in blocking solution (o/n 4°C). Following four washes in PBSTr samples were incubated with fluorescence-labeled secondary antibodies in blocking solution

(2h, RT) followed by three washes in PBSTr, one wash in PBSTr containing 2 μg/ml DAPI and three washes in PBSTr prior to mounting in Vectashield with DAPI (H-1200). Samples were imaged the following day on an Olympus Fluoview FV1000 confocal microscope. Antibodies are listed in the Key Resources Table.

Colony forming assay

675 single ESCs (SL) of three independent clones per genotype were seeded on gelatin coated 6cm dishes and fed daily. On day 7 colonies were washed with PBS, fixed with 70% ethanol (10min, room temperature) stained with Giemsa stain (GIBCO) for 15min at RT, washed extensively with tap water and air-dried. Colony area surface was measured at day 5 of colony formation using Fiji (Schindelin et al., 2012).

Alkaline phosphatase staining

600 single ESCs (SL) from three independent clones per genotype were seeded. The following day, medium was exchanged to medium without or with LIF and replaced daily. On d5 cells were washed in PBS, fixed in 4% PFA (1min at RT), washed in TBST, stained with alkaline phosphatase staining solution (Millipore, Cat#SCR004) for 15 min at RT, washed in TBST and imaged. Differentiated, mixed and undifferentiated colonies were scored and counted.

QUANTIFICATION AND STATISTICAL ANALYSIS

RT-PCR

Statistical significance between Smad2/3 DKO and WT samples was determined using the R function wilcox.test with default parameters. Significance levels are denoted by * for $p < 0.05$ and ** for $p < 0.01$.

Microarray analysis

Bead-Station data were extracted using the Gene Expression Analysis Module V1.6.0 of GenomeStudio V2009.2 (Illumina) and imported into an R-based Illumina pre-processing module (Eijssen et al., 2015). Hierarchical clustering identified three outlier samples in ESCs that were excluded from subsequent analysis. Differential probe expression was determined using an R-based statistical analysis module (Eijssen et al., 2015) with raw *p* values compared to averaged WT signal at the same time-point. Unique ILMN_GENE gene identifiers of probes with significantly different expression ($p < 0.05$ and fold change > 1.5) were identified and were analyzed using Venn diagram overlaps (Hulsen et al., 2008). For WT top expressed genes, d3 EB datasets were compared to averaged ESC datasets. Heatmaps of log2FC values for individual clones were made using Java TreeView (Saldanha, 2004).

ATAC-seq, ChIP-seq and RNA-seq analysis

Paired-end reads for ATAC-seq and ChIP-seq were aligned to the mouse mm10 genome using bowtie2 (Langmead and Salzberg, 2012) with the "–no-mixed" and "–no-discordant" options. Non-uniquely mapping reads and reads mapping to a custom "blacklist" of artificially high regions of the genome were discarded. For RNA-seq, reads were initially aligned using bowtie2 against the rRNA genomic sequence (GenBank: BK000964.3) to filter out rRNA fragments, prior to alignment against the mm10 genome using the STAR RNA-seq aligner with default parameters (Dobin et al., 2013) PCR duplicates were removed using Samtools (Li et al., 2009). Biological replicates were randomly downsampled to contain the same number of reads for each individual replicate, and merged to create a representative genome track using DANPOS2 (Chen et al., 2013) for ATAC-seq samples and MACS2 (Zhang et al., 2008) for ChIP-seq and genomecov from bedtools (Quinlan and Hall, 2010) for RNA-seq. Genome coverage tracks were visualized using the UCSC Genome Browser.

ATAC hypersensitive sites in both WT and SMAD2/3 null EpiLCs were identified using the DANPOS2 dpeak function with biological triplicates and a Tn5 genomic DNA control. Significant changes in ATAC-seq datasets were identified using the DiffBind package (Stark and Brown, 2011). For RNA-seq, the mm10 refGene gene bodies were annotated with biological replicate read counts and gene expression changes were identified with DESeq2 (Love et al., 2014). For both DiffBind and DESeq2, a FDR < 0.05 and a fold change > 1.5 was deemed a significant change. To link differential gene expression with Smad2/3 ChIP-seq signal in ESCs and EBs (Wang et al., 2017), we called Smad2/3 peaks using DANPOS2, annotated peaks with the closest RefSeq TSS using HOMER (Heinz et al., 2010) and excluded peaks > 50kb away from RefSeq TSSs.

Changes in ATAC-seq were visualized using heatmaps produced using HOMER and Java TreeView. GREAT was used to identify the distance of peaks to known TSS (McLean et al., 2010). To identify accessibility changes during the ESC to EpiLC transition, we used ATAC-seq data from Simon et al. (2017) with DiffBind as above. To compare differential chromatin accessibility with changes in nearby gene expression, we used HOMER to identify the transcriptional start sites (TSS) nearest to sites with differential chromatin accessibility and visualized the log2FC gene expression changes in RNA-seq data from Smad2/3 DKO EpiLCs in comparison to WT EpiLCs as a heatmap. Differentially accessible sites in Smad2/3 DKO EpiLCs were annotated with published p300, H3K27ac, H3K4me1, Smad2/3, Smad1, Oct4 and Otx2 ChIP-seq data (Buecker et al., 2014; Morikawa et al., 2016; Wang

et al., 2017). Enrichment of transcription factor motifs in differentially accessible ATAC-seq peaks was performed using the Analysis of Motif Enrichment (AME) feature in the MEME suite (McLeay and Bailey, 2010) with a background control of unaffected ATAC-seq peaks.

DATA AND SOFTWARE AVAILABILITY

The accession number for the data reported in this paper is GEO: GSE110164 (https://www.ncbi.nlm.nih.gov/geo/query/acc.cgi?acc=GSE110164).

Cell Reports, Volume *24*

Supplemental Information

Combinatorial Smad2/3 Activities Downstream of Nodal Signaling Maintain Embryonic/Extra-Embryonic Cell Identities during Lineage Priming

Anna D. Senft, Ita Costello, Hamish W. King, Arne W. Mould, Elizabeth K. Bikoff, and Elizabeth J. Robertson

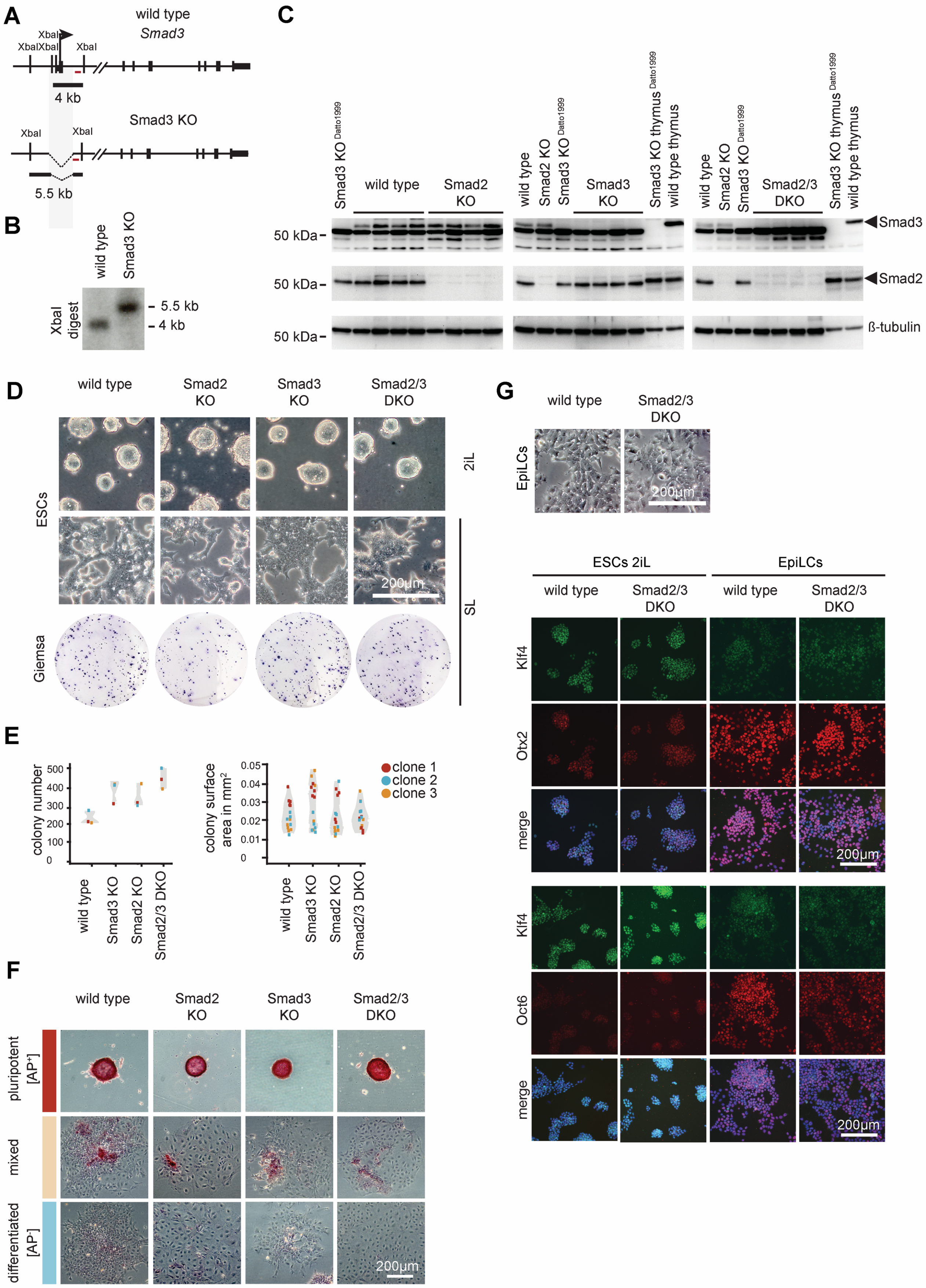

Figure S1
related to Figure 1

Generation and characterization of Smad2/3 DKO, Smad3 KO and Smad2 KO ESCs and Smad2/3 DKO EpiLCs

A. Smad2/3 DKO and Smad3 KO ESC lines were obtained by targeting Smad2 KO and WT ESC lines using the *Smad3* deletion strategy shown. The shaded area indicates the genomic region of mouse *Smad3* around exon 1 removed by CRISPR/Cas9 nickase technology. Red bar and thin black lines depict the 3' Southern probe and XbaI restriction sites used for genotyping the deletion in (B). See Key Resources Table and Table S4 for sequences for sgRNAs and Southern probe.
B. Southern blot to screen for $Smad3^{\Delta exon1/\Delta exon1}$ and Smad2 KO;$Smad3^{\Delta ex1/\Delta ex1}$ (hereafter referred to as Smad3 KO and Smad2/3 DKO) ESC lines with the 3' probe depicted in (A) on XbaI-digested genomic DNA. Homozygous targeted clones contain a single band at 5.5 kb compared to 4 kb in WT *Smad3*.
C. Western blot of Smad2/3 DKO, Smad3 KO as well as parental Smad2 KO and WT ESC lines used in study with antibodies specific to Smad3, Smad2 and β-tubulin. The recognition site of the Smad3 antibody lies in the region most divergent between the highly homologous Smad2 and Smad3 proteins and is - on the genomic level - unaffected by the deletion strategy used. Conventionally derived $Smad3^{tm1Xfw/tm1Xfw}$ ESCs as well as thymus isolated from $Smad3^{tm1Xfw/tm1Xfw}$ male mice (Datto et al. 1999) were used as controls. Note that prominent bands in Smad3 blots represent unspecific background that is present in all ESC samples but absent in thymus lysates.
D. Morphology of WT, Smad2 KO, Smad3 KO and Smad2/3 DKO ESCs grown under 2iL and SL conditions. Giemsa staining of ESC colonies grown for 7 days following plating of 675 ESCs (SL).
E. Violin plots depicting colony number from cultures of three independent ESC lines per genotype (left) and colony surface area of 5 colonies for each independent ESC line (right).
F. Representative images of pluripotent (red), mixed (beige) and differentiated (blue) colonies after alkaline phosphatase (AP) staining on WT, Smad2 KO, Smad3 KO and Smad2/3 DKO ESC colonies (SL) grown for five days in the presence or absence of LIF. Related to Figure 1C.
G. Brightfield images and immunofluorescence staining of WT and Smad2/3 DKO ESCs (2i) and EpiLCs using Klf4 together with Otx2 or Oct6-specific antibodies and counterstained with DAPI.

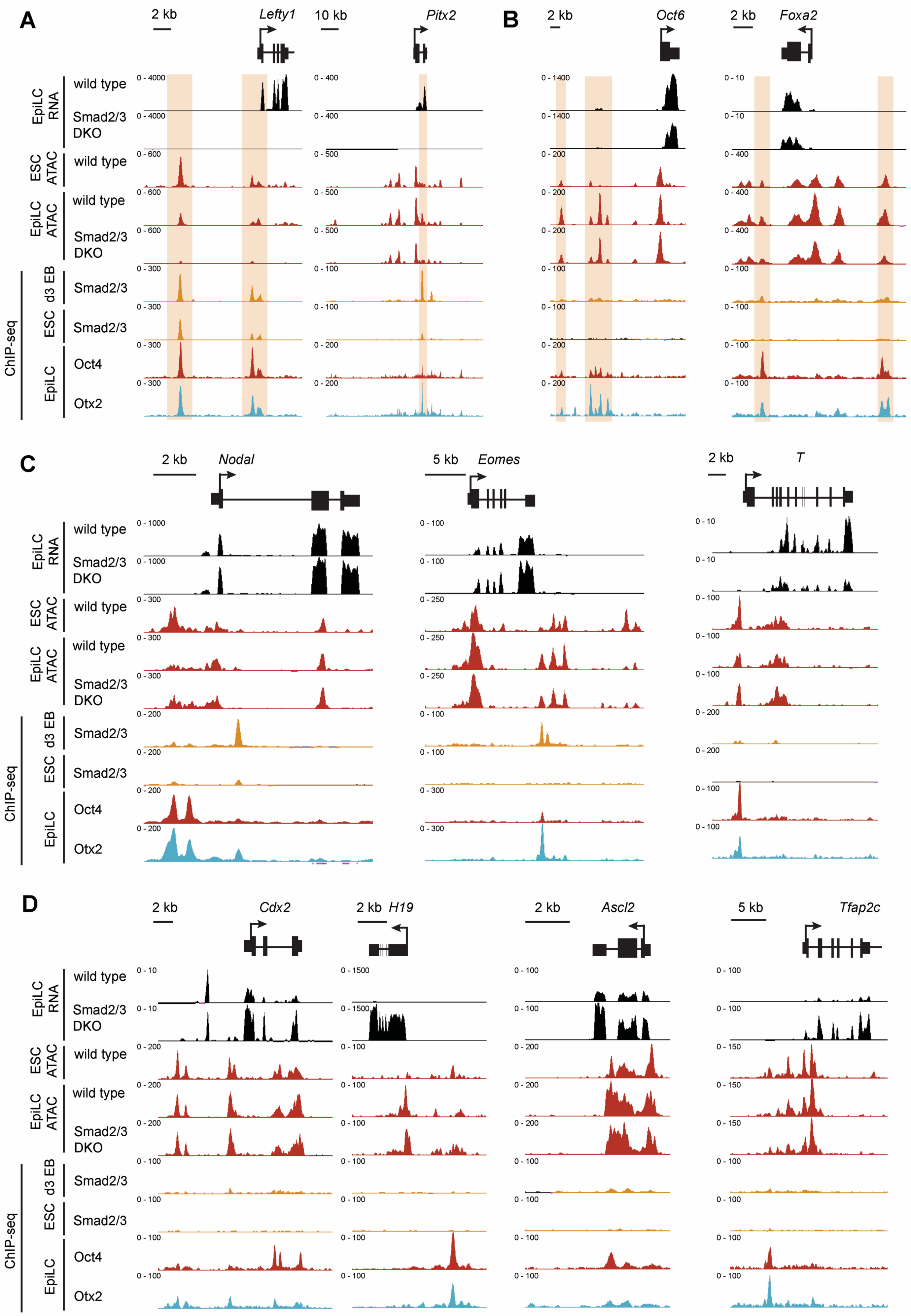

Figure S2
related to Figure 2

Smad2/3 alters chromatin accessibility at Nodal-dependent and priming marker genes but not at embryonic and extra-embryonic differentiation genes in EpiLCs

A-D. Genome browser screenshots of RNA-seq and ATAC-seq tracks in Smad2/3 DKO and WT EpiLCs at loci of Nodal target genes (A), primed pluripotency genes (B), early differentiation markers (C) and extra-embryonic marker genes (D). ChIP-seq tracks of Smad2/3 occupancy in ESCs and d3 EBs (Wang et al. 2017) and Oct4 and Otx2 occupancy in EpiLCs (Buecker et al., 2014) and ATAC-seq signal in ESCs (Simon et al., 2017) (D) are also shown.

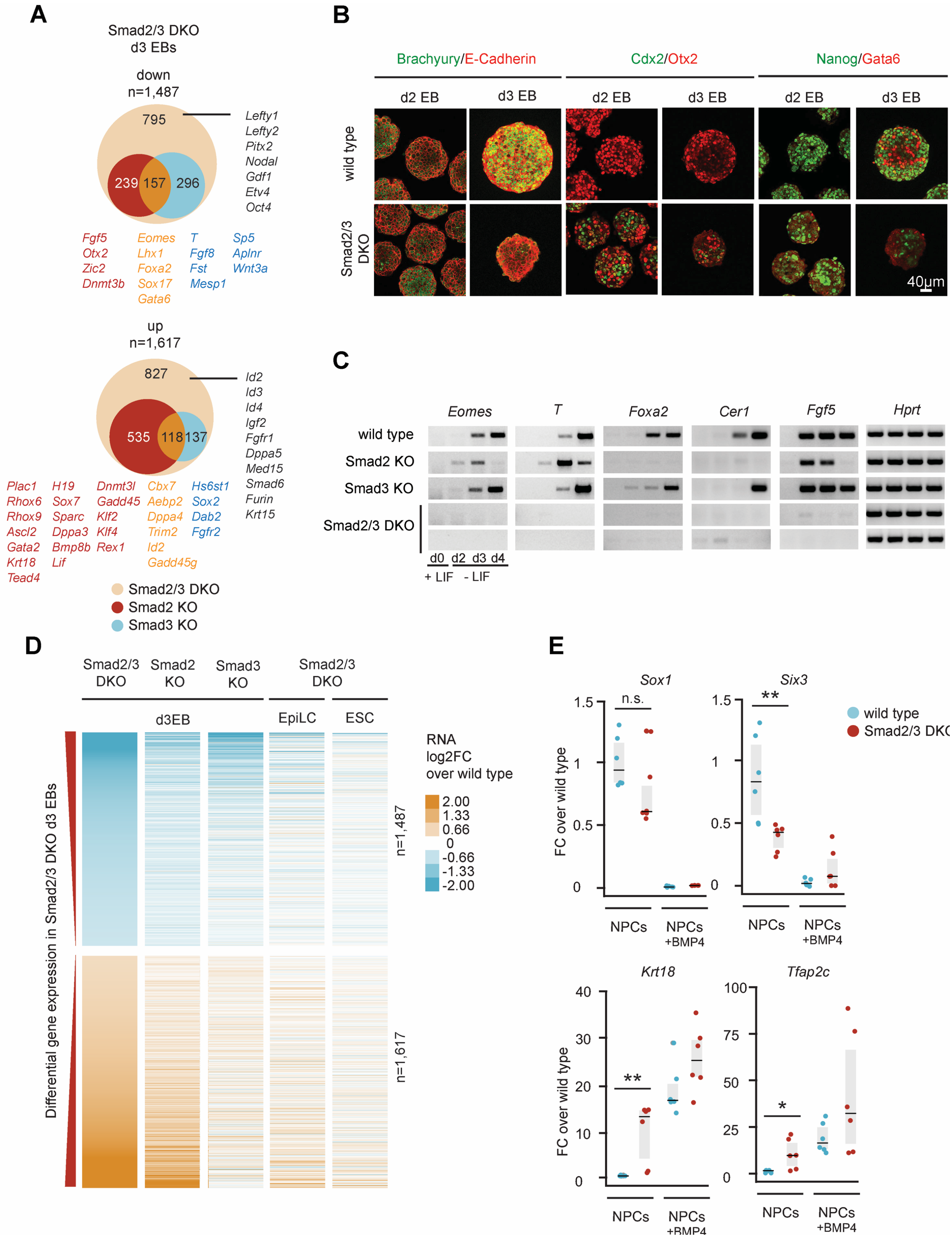

Figure S3
related to Figure 3

Combinatorial Smad2 and Smad3 activities mediate embryonic cell fate allocation and repression of extra-embryonic gene expression

A. Venn diagrams showing the overlap between significant changes in gene expression in Smad2 KO, Smad3 KO and Smad2/3 DKO d3 EBs relative to WT d3 EBs as determined by microarray profiling (n=4). Genes unique to Smad2 KO or Smad3 KO EBs were omitted. A list of all deregulated genes is shown in Table S1.
B. Immunofluorescence staining of WT and Smad2/3 DKO d2 and d3 EBs with antibodies specific to Brachyury and E-Cadherin (left), Otx2 and Cdx2 (centre) and Nanog and Gata6 (right).
C. Validation of reduced gene expression of selected down-regulated genes during EB differentiation of WT, Smad2 KO, Smad3 KO and Smad2/3 DKO ESCs by OneStep RT-PCR. *Hprt* expression is used as a control.
D. Heatmap showing the log2FC in expression of differentially expressed genes in Smad2/3 DKO d3 EBs as compared to WT d3 EBs and their expression in Smad2 KO and Smad3 KO d3 EBs, Smad2/3 DKO EpiLCs and ESCs relative to their respective WT controls.
E. qRT-PCR measurement of gene expression changes in Smad2/3 DKO d5 NPCs (n=3) grown in the absence or presence of BMP4 (5ng/ml) using primers specific to *Sox1*, *Six3*, *Krt18* and *Tfap2c* (encoding AP2γ) normalized to *Gapdh* expression. *$p<0.05$, **$p<0.01$.

Table S4 related to Figures S1 and S3

List of primers used in this study including PCR and Southern probe genotyping primers, OneStep RT-PCR and qRT-PCR primers.

Category / Gene	forward	reverse
Genotyping		
Smad3 tm1Xfw	CTCCAGAGTTAAAAGCGAAGTTCG	rev1: AAAATGCTGCACGGAAGCCAGGTC<br>rev2: ATTTGTCACGTCCTGCACGACG
Smad2 Robm1	TATTGCTGAAGAGCTTGGCG	rev1: TGGCCTTAGAAAAGTTAATTGCCC<br>rev2: GCAAACCACAGCACACAACA
Southern blot probe		
Smad3 3' exon 1	CAGGCGATGGTTCTGTCCTT	GCCAGGGCAGGATTCAAGAT
OneStep RT-PCR		
Eomes	TGTTTTCGTGGAAGTGGTTCTGGC	AGGTCTGAGTCTTGGAAGGTTCATTC
T	AACTTTCCTCCATGTGCTGAGAC	TGACTTCCCAACACAAAAAGCT
Foxa2	TGGCTGCAGACACTTCCTACT	CAACATCAGTACAACCCTCTGGT
Cer1	GGAGGAAGCCAAGAGGTTCT	GTCTTCATGGGCAATGGTCT
Fgf5	TTGCGACCCAGGAGCTTAAT	CTACGCCTCTTTATTGCAGC
Hprt	GCTGGTGAAAAGGACCTCT	CACAGGACTAGAACACCTGC
qRT-PCR		
Sox1	GTGACATCTGCCCCCATC	GAGGCCAGTCTGGTGTCAG
Six3	CCGGAAGAGTTGTCCATGTTC	CGACTCGTGTTTGTTGATGGC
Krt18	CAGCCAGCGTCTATGCAGG	CTTTCTCGGTCTGGATTCCAC
Tfap2c	GCCGGACGCCATGTTGTGGA	ACCCCGGTGTGCGAGAGAGG
Gapdh	TGCACCACCAACTGCTTAGC	GGCATGGACTGTGGTCATGAG
